# Domain-specific p53 mutants activate EGFR by distinct mechanisms exposing tissue-independent therapeutic vulnerabilities

**DOI:** 10.1038/s41467-023-37223-3

**Published:** 2023-03-28

**Authors:** Teresa Lai Fong Ho, May Yin Lee, Hui Chin Goh, Germaine Yi Ning Ng, Jane Jia Hui Lee, Srinivasaraghavan Kannan, Yan Ting Lim, Tianyun Zhao, Edwin Kok Hao Lim, Cheryl Zi Jin Phua, Yi Fei Lee, Rebecca Yi Xuan Lim, Perry Jun Hao Ng, Ju Yuan, Dedrick Kok Hong Chan, Bettina Lieske, Choon Seng Chong, Kuok Chung Lee, Jeffrey Lum, Wai Kit Cheong, Khay Guan Yeoh, Ker Kan Tan, Radoslaw M. Sobota, Chandra S. Verma, David P. Lane, Wai Leong Tam, Ashok R. Venkitaraman

**Affiliations:** 1 Disease Intervention Technology Lab (DITL), Institute of Molecular and Cell Biology, Agency for Science Technology and Research (A*STAR), Singapore, Singapore; 2 Cancer Science Institute of Singapore, National University of Singapore, Singapore, Singapore; 3 Genome Institute of Singapore, Agency for Science, Technology and Research (A*STAR), Singapore, Singapore; 4 Singapore Institute of Technology, Singapore, Singapore; 5 Bioinformatics Institute, Agency for Science, Technology and Research (A*STAR), Singapore, Singapore; 6 Functional Proteomics Laboratory, Institute of Molecular and Cell Biology, Agency for Science, Technology and Research (A*STAR), Singapore, Singapore; 7 SingMass – National Mass Spectrometry Laboratory, Institute of Molecular and Cell Biology, Agency for Science, Technology and Research (A*STAR), Singapore, Singapore; 8 Nuffield Department of Surgical Sciences, University of Oxford, Oxford, UK; 9 Division of Colorectal Surgery, University Surgical Cluster, National University Health System, Singapore, Singapore; 10 Department of Surgery, Yong Loo Lin School of Medicine, National University of Singapore, Singapore, Singapore; 11 Department of Pathology, National University Health System, Singapore, Singapore; 12 University Surgical Cluster, National University Health System, Singapore, Singapore; 13 School of Biological Science, Nanyang Technological University, Singapore, Singapore; 14 Department of Biological Science, National University of Singapore, Singapore, Singapore; 15 Department of Biochemistry, Yong Loo Lin School of Medicine, National University of Singapore, Singapore, Singapore; 16 NUS Center for Cancer Research, Yong Loo Lin School of Medicine, National University of Singapore, Singapore, Singapore

**Keywords:** Oncogenes, Growth factor signalling

## Abstract

Mis-sense mutations affecting *TP53* promote carcinogenesis both by inactivating tumor suppression, and by conferring pro-carcinogenic activities. We report here that p53 DNA-binding domain (DBD) and transactivation domain (TAD) mis-sense mutants unexpectedly activate pro-carcinogenic epidermal growth factor receptor (EGFR) signaling via distinct, previously unrecognized molecular mechanisms. DBD- and TAD-specific *TP53* mutants exhibited different cellular localization and induced distinct gene expression profiles. In multiple tissues, EGFR is stabilized by TAD and DBD mutants in the cytosolic and nuclear compartments respectively. TAD mutants promote EGFR-mediated signaling by enhancing EGFR interaction with AKT via DDX31 in the cytosol. Conversely, DBD mutants maintain EGFR activity in the nucleus, by blocking EGFR interaction with the phosphatase SHP1, triggering c-Myc and Cyclin D1 upregulation. Our findings suggest that p53 mutants carrying gain-of-function, mis-sense mutations affecting two different domains form new protein complexes that promote carcinogenesis by enhancing EGFR signaling via distinctive mechanisms, exposing clinically relevant therapeutic vulnerabilities.

## Abstract (web summary)

Here the authors identify distinct mechanisms by which domain-specific p53 mutations activate cancer cell growth via the epidermal growth factor receptor (EGFR). These mechanisms affect sensitivity to EGFR inhibitors, opening avenues for targeted therapy.

## Introduction

Diverse mis-sense mutations found in all regions of the *TP53* gene frequently engender mutant proteins that lose tumor suppressive functions, and/or gain new oncogenic properties. The degree of loss of tumor suppressive functions, versus the context-dependent manifestation of gain-of-function (GOF) phenotypes, is highly variable between p53 mutants^1–3^, and the molecular mechanisms underlying these differences are unknown. This diversity in the form and function of mutant p53 proteins challenges our fundamental understanding of how mutant p53 influences tumor development, and impedes therapeutic targeting^4^. Hence, uncovering GOF mechanisms amongst and between domain-specific mutants that functionally influence tumor development is vital.

Most *TP53* mutations commonly identified in cancer occur within the DNA-binding domain (DBD) at so-called ‘hotspot’ residues. These extensively studied DBD hotspot mutations are mainly categorized into DNA contact mutations that affect p53 binding to DNA, or conformational mutations which destabilize the folding of the DBD region^5,6^. One common GOF property of DBD mutants is their ability to bind novel, non-canonical promoter sites, and regulate genes through association with other transcription factors like ETS2^7^, E2F, HIF-1α, SMAD, and NF-kB^5^. However, not all DBD mutants share this particular GOF property and even if they do, gene subsets and interaction partners may differ. Structural similarities amongst mutant proteins do not reliably correlate with shared GOF properties^8^.

Many *TP53* mutations, often of unknown functional significance, occur in domains other than the DBD. One such domain, critical for p53 tumor suppression, is the transactivation domain (TAD), which often serves as the interaction site for several transcriptional regulators and chromatin modifiers. Recent large-scale genomic studies of multiple cancer types have revealed that TAD mutant tumors make up a significant clinical minority. Residues Leu22 (L22), Trp23 (W23), Leu 25 (L25), Leu26 (L26), Trp53 (W53), and Phe54 (F54) have been identified as important for transactivation function and tumor suppression^9,10^. However, little is known about how TAD mutants might differ from DBD mutants in terms of potential GOF properties. It is also unclear if such GOF properties depend on overall structural changes in the mutant protein, or instead, are domain-specific. Moreover, some TAD mutants seemingly retain the ability to regulate either apoptosis or cell cycle processes, while others lose all transcriptional abilities^11^, underlying variable loss of their tumor suppressive properties. Sequencing analyses have also uncovered differences in mutational burdens and immunological profiles of DBD and TAD tumors^12–15^. Again, the molecular mechanisms underlying these differences are unclear.

Here, we report distinct, previously unrecognized GOF effects of TAD and DBD mutations in *TP53* that enhance epidermal growth factor receptor (EGFR) activity. EGFR amplification often co-occurs with *TP53* mutations; notably, p53 mutations affect EGFR therapy stratification and clinical outcomes^16,17^. Canonical, ligand-dependent EGFR signaling has been well characterized^18^. Ligand binding activates EGFR dimerization and the transduction of multiple signaling pathways, including the PI3K-AKT-mTOR, Ras/MAPK, and PLC-PKC axes. EGFR also mediates kinase-independent, pro-survival functions under various conditions of stress, through its interactions with sodium/glucose co-transporter 1 (SGLT1) and fatty acid synthase (FASN), to maintain glucose uptake and de novo fatty acid synthesis respectively. Non-canonical functions of EGFR in the nucleus have also been studied extensively^19^. Apart from phosphorylating nuclear proteins such as histones, PCNA^20^, STAT3, and ATM^21^, nuclear EGFR can also transactivate genes such CycD1^22,23^, Aurora-A, c-Myc^24^, B-Myb, STAT1^25^, and iNOS, through binding of STATs and E2F1^26^.

We show in this paper that domain-specific *TP53* mutants exhibit distinct, tissue-independent cellular activities that modulate key growth signaling pathways. Specifically, we find that TAD and DBD mutants modulate canonical and non-canonical functions of EGFR through their common ability to bind and stabilize EGFR. TAD mutants promote EGFR-mediated signaling through enhancing EGFR interaction with AKT via a known mutant p53 interactor, DDX31. In contrast, DBD mutants promote EGFR activity in the nucleus by blocking EGFR’s interaction with SHP1. The effects of mutant p53 reported here differ from ‘classical’ transactivation of novel gene targets, but rather, are mediated through stabilization or disruption of distinct protein signaling complexes. These previously unrecognized mechanisms of domain-specific mutant p53 are significant not only in furthering our understanding of how different classes of *TP53* mutants exert pro-carcinogenic GOF effects, but may also guide clinical stratification, and the development of therapeutic approaches that exploit common dependencies in tumors wherein p53 and/or EGFR function have been perturbed.

## Results

### Cytosolic stabilization of TAD mutants promotes distinct, domain-specific protein interactions

**Fig. 1:**
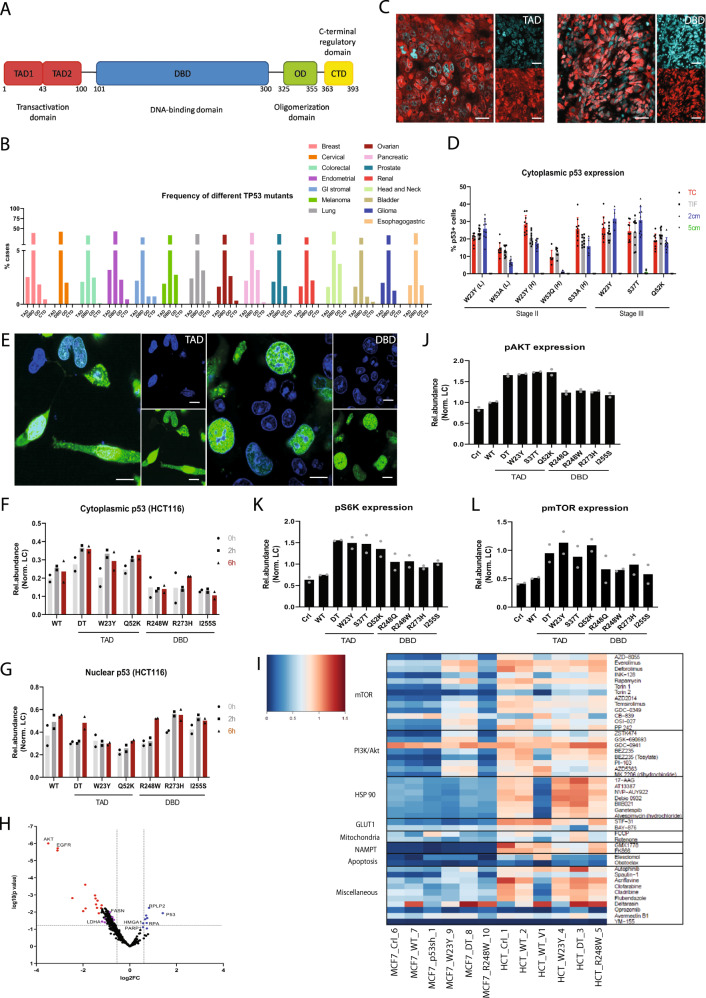
TAD and DBD-mutant colorectal tumors and cells bear unique molecular differences. **A** Domain structure of p53. **B** Frequency of different domain-specific *TP53* mutations as reported in tissue-specific cancers. **C** Representative immunofluorescence staining of p53 (red) in colorectal tumor tissues. DAPI staining (cyan) shows nuclei. Scale bar = 30 μm. **D** Quantification of the percentage of cells with cytosolic p53 staining in TAD mutant colorectal tumors (Mean ± SEM). Different regions of the tumor (TC: tumor core; TIF: tumor invasive front) and surrounding normal tissue (2 cm and 5 cm from the tumor invasive front) were analyzed. Each mutation represents an individual patient. **E** Representative immunofluorescence staining of p53 (green) in HCT116 cells expressing *TP53* mutants. DAPI staining shows nuclei. *n* = 3 independent experiments. Scale bar = 10 μm. **F**, **G** Quantification of p53 protein turnover in HCT116 (**F**) cytosolic and (**G**) nuclear cellular fractions following treatment with cycloheximide. *n* = 2. **H** Volcano plot of IP-MS analysis of *TP53* mutant expressing HCT116 cells. Each point represents a p53 protein interactor with adjusted p values on the y axis and log fold change in abundance on the x axis. Proteins with negative log fold change in abundance are relatively enriched in TAD mutant cells (red and purple points) while those with positive log fold change in abundance are relatively enriched in DBD-mutant cells (blue points). **I** Heat map indicating differential sensitivities of HCT116 and MCF7 cells expressing WT and mutant p53 to a customized library of small molecule inhibitors. Compounds are grouped according to signaling and metabolic pathways and/or molecular function. *n* = 2. HCT_WT_V1 contains endogenous p53 while HCT_WT_2 is a p53 null line where WT p53 has been reintroduced. Cell lines expressing the TAD mutants W23Y and DT and DBD-mutant R248W were used. **J**–**L** Quantification of protein levels of **J** pAKT, **K** pS6K and **L** pmTOR in HCT116 cells expressing WT and mutant p53. *n* = 2. Source data are provided as a Source Data file.

In the IARC *TP53* Mutation Database, about 95% of oncogenic *TP53* mutations occur within the DBD (Fig. 1A and S1A, B). Frequent, non-random nucleotide alterations have been identified in ‘hotspot’ codons at positions R175, R248, and R273. Analysis of mutational data reported in the MSK-IMPACT clinical cohort revealed that DBD-mutant tumors constitute about half of all cases while TAD mutant tumors constitute a significant clinical minority across major tissue types (Fig. 1B).

To better characterize molecular differences between DBD and TAD mutant tumors, we analyzed clinical samples derived from a cohort of colorectal cancer (CRC) patients recruited at the National University Hospital, Singapore (Supplementary Fig. 1C). Mutational status and loss of the wildtype (WT) allele were verified by sequencing. Tumors which lack detectable p53 protein and RNA were categorized as knockout (KO) and further verified by sequencing. Strikingly, we observed both cytosolic and nuclear localization of TAD mutant p53, while DBD p53 was largely confined to the nucleus (Fig. 1C). A further dissection of tumors into different spatial regions – tumor core (TC), tumor invasive front (TIF) and morphologically normal tissue (2 cm and 5 cm from the TC) – revealed that cytosolic TAD mutant p53 was present in morphologically normal tissues up to 2 cm from the TC (Fig. 1D). By contrast, in DBD-mutant tumors, nuclear expression of mutant p53 was restricted only within TC and TIF (Supplementary Fig. 1D). Interestingly, elevated levels of cytosolic TAD mutant p53 were associated with late-stage tumors. Furthermore, early-stage TAD tumors with elevated cytosolic mutant p53 expression exhibited similar molecular characteristics as late-stage TAD tumors, such as increased levels of Ki67 and γH2AX (Supplementary Fig. 1E, F). This correlation was observed regardless of the degree of risk (H: high; L: low) of disease recurrence and/or metastasis that was clinically prognosticated in Stage II CRC patients based on histopathological parameters.

To further investigate the molecular mechanisms by which TAD and DBD mutants may influence tumor growth, we generated HCT116, H1299 and MCF7 cells that stably expressed our mutants of interest. We also included a previously characterized TAD mutant containing mutations in both TAD domains (LW22/23QS, WF53/54QS) known as the DT mutant, which has been reported to be transcriptionally inactive^10,27^. We observed consistent cytosolic stabilization of TAD mutants in cell lines from different tissues (Fig. 1E–G and Supplementary Fig. 1G–I). Using a cellular thermal shift assay (CETSA), we determined that cytosolic stabilization of TAD mutants was accompanied by corresponding thermal stabilization with an increase in the melting temperature (T50/’IC50’) of the mutant protein (Supplementary Fig. 1J–M). Hence, differential intracellular stabilization of DBD and TAD mutants serves as a domain-specific characteristic that may differentiate these two classes of p53 mutants. In comparison, the use of transcriptional capability to distinguish TAD and DBD mutants is less effective, given the variability between mutants and tissue-specific cell lines as shown by our reporter assay and qRT-PCR analysis of p53 target genes (Supplementary Fig. 2A–I).

We next assessed the in vivo ability of TAD and DBD mutant cells to form tumors. We found that TAD mutant MCF7 cells were as proficient, if not better, at tumor formation and outgrowth compared to DBD mutant cells (Supplementary Fig. 2J). Cytosolic expression of TAD mutants in MCF7 and HCT116 xenograft tumors (Supplementary Fig. 2K, L) further recapitulated observations in patient CRC tumors and in vitro. The consistent cellular localization patterns of TAD and DBD mutants in patient and xenograft tumors and stable HCT116 and H1299 cells suggest that this is a domain-specific property with potential implications for distinct molecular mechanisms. We used colony-forming assays as a surrogate^28,29^ for the tumor-forming potential of TAD and DBD mutants. We found that the presence of mutant p53 significantly improved colony formation in three separate cellular systems over control (p53 null) cells (Supplementary Fig. 2M–O). These findings suggest that the properties conferred by TAD and DBD mutants may be biologically significant in carcinogenesis.

Since mutant p53 proteins can gain novel protein interactors through altered protein folding and/or cellular (mis)localization, we conducted immunoprecipitation (IP) experiments in combination with mass spectrometry (MS) to identify mutant p53 interacting partners. We identified AKT, EGFR, fatty acid synthase (FASN), and lactate dehydrogenase A (LDHA) as being relatively enriched amongst TAD mutants, whereas HMGA1 and PARP1 were relatively enriched amongst DBD mutants (Fig. 1H). Reactome pathway analysis of protein hits showed preferential interactions between TAD mutants and proteins involved in signal transduction, metabolism, and cellular compartmentalization, while DBD mutants demonstrated increased interactions with typically nuclear proteins involved in transcriptional regulation, DNA repair and replication and cell cycle control (Supplementary Fig. 2Q, R). We validated selected IP-MS targets with co-IP assays using a panel of TAD and DBD mutants (Supplementary Fig. 2P), showing that these are indeed bona fide interactors gained by mutant p53.

Collectively, our findings indicate that mutations affecting the TAD or DBD of p53 result in distinct cellular localization patterns and protein partners that may contribute to the molecular characteristics of cancers that carry them.

### TAD mutations lead to dependence on PI3K/AKT/mTOR signaling pathways

We therefore tested whether the unique protein interactions of p53 TAD versus DBD mutants, might engage different signaling pathways and mechanisms that give rise to distinctive pro-survival or pro-carcinogenic effects.

To this end, we conducted a screen using a customized library of 303 small molecule inhibitors that target signaling and metabolic pathways, in order to identify and validate signaling pathways upon which p53 TAD versus DBD mutant cells uniquely depend upon for growth or survival (Fig. 1I and Supplementary Fig. 3A). We found that selective inhibition of PI3K/AKT/mTOR signaling and biosynthesis consistently reduced cell viability more markedly in TAD mutant cells versus DBD mutant counterparts (Fig. 1I and Supplementary Fig. 3A). In both HCT116 and H1299 cells, IC50 concentrations for PI3K, AKT and mTOR inhibitors were lower in TAD-expressing cells (Supplementary Fig. 3B–D, F–H). Conversely, IC50s of EGFR inhibitors were lower in DBD-expressing cells (Supplementary Fig. 3E, I). Western blot analysis revealed increased levels of phosphorylated AKT, S6K, ERK and mTOR in TAD mutant cells (Fig. 1J–L and Supplementary Fig. 3J–M), supporting our observation that TAD mutant cells show increased sensitivity to inhibitors of these pathways. Collectively, these data suggest that p53 TAD versus DBD mutant cells depend on distinct mechanisms for their survival or growth, and exhibit distinctive patterns of activation in intracellular signaling pathways. Our results raise the possibility that these differences could be used to differentiate between domain-specific p53 mutants.

### Mutant p53 stabilizes EGFR, promoting cytosolic, and nuclear signaling

**Fig. 2:**
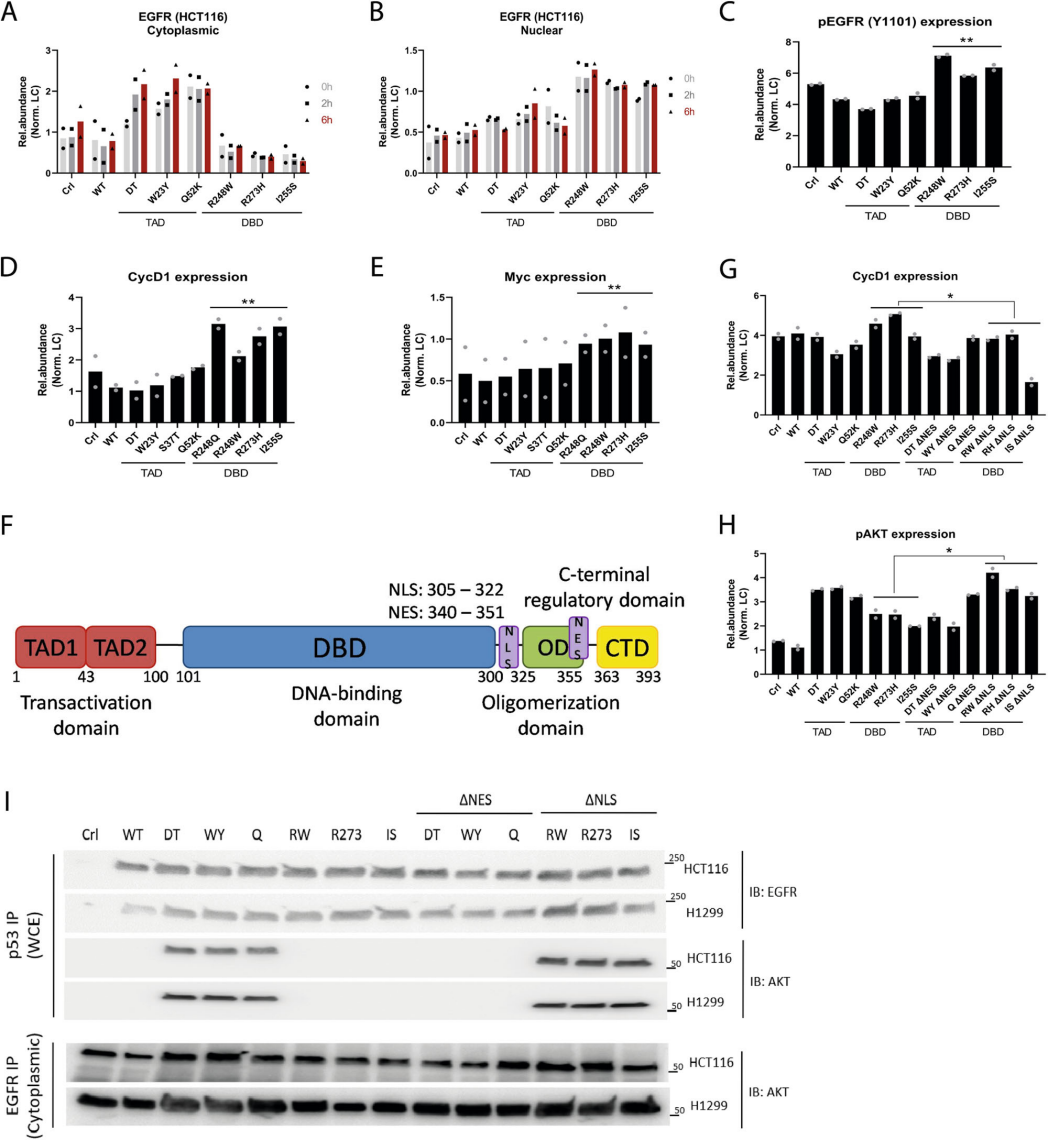
TAD and DBD mutant p53 stabilize EGFR and promote different facets of EGFR function. **A**, **B** Quantification of EGFR protein turnover in HCT116 **A** cytosolic and **B** nuclear cellular fractions following treatment with cycloheximide. *n* = 2. **C**–**E** Quantification of **C** pEGFR (Y1101), **D** Cyclin D1 and **E** c-Myc protein levels in HCT116 cells expressing WT and mutant p53. Statistical tests performed on TAD versus DBD. *n* = 2. **F** Domain structure of p53 with nuclear localization (NLS) and export signal (NES) residues indicated. **G**, **H** Quantification of **G** Cyclin D1 and **H** pAKT protein levels in HCT116 cells expressing WT and mutant p53. *n* = 2. **I** Protein levels of EGFR and AKT pulled down with p53 and/or EGFR in HCT116 and H1299 cells expressing WT and mutant p53. *n* = 2. Two-tailed unpaired Student’s *t* test was performed on TAD (*n* = 3 independent mutants) versus DBD (*n* = 3 independent mutants). (*p < 0.05, **p < 0.01, ***p < 0.001, ****p < 0.0001). Source data are provided as a Source Data file.

Despite these differences, we observed that both DBD and TAD mutant forms of p53 interact with the EGFR. The EGFR-AKT-PI3K-mTOR axis plays a central role in cellular growth and metabolism and is frequently altered in cancer. We therefore investigated whether TAD versus DBD forms of mutant p53 affected EGFR protein stability or signaling. A pulse-chase assay using cycloheximide combined with cellular fractionation in HCT116 and H1299 cells showed that the half-life of cytosolic and nuclear EGFR were increased in TAD and DBD mutant cells respectively (Fig. 2A, B and Supplementary Fig. 4A–C). Increased stabilization of cytosolic EGFR by TAD mutants was consistent with our aforementioned observations of increased signaling along the EGFR-AKT-PI3K-mTOR axis. By contrast, increased stabilization of nuclear EGFR in DBD mutants correlated with increased levels of phosphorylated EGFR (Y1101), which has been implicated in EGFR nuclear trafficking and retention (Fig. 2C and Supplementary Fig. 4D). Levels of ligand-activated phosphorylated EGFR (Y1068), which can be found in both the cytosol and nucleus, were comparable between both classes of mutants in HCT116 cells, but elevated in DBD-mutant expressing H1299 cells (Supplementary Fig. 4E, H).

Nuclear EGFR has been reported to function as a transcriptional co-activator for genes such as Cyclin D1 and c-Myc^30,31^. Consistent with this function, we found that increased nuclear stabilization and phosphorylation of EGFR in DBD mutant cells was accompanied by upregulation of Cyclin D1 in both HCT116 and H1299 cells, and of c-Myc in HCT116 cells (Fig. 2D, E and Supplementary Fig. 3M and 4F–G). The E2F family of transcription factors, especially E2F1, regulates a variety of cell cycle targets including cyclins and the MyB family of proteins^32,33^. E2F1 is also a known interactor of mutant p53 and both mutant p53 and EGFR can ‘hijack’ E2F1 transcriptional targets^5,7^. Using pulldown assays on nuclear extracts, we observed an increase in interaction between EGFR and E2F1 in DBD mutant cells (Supplementary Fig. 4H), further supporting the observed increase in Cyclin D1 and c-Myc. Collectively, our findings suggest that domain-specific *TP53* mutants differentially promote EGFR stability in the cytosol (TAD mutants) versus the nucleus (DBD mutants), and in turn, stimulate specific functions of EGFR.

### Forced mis-localization of DBD mutants to the cytosol enhances EGFR-AKT signaling

Mutant p53 is known to interact with a variety of proteins in a context- and structure-specific manner. We therefore asked if forced nuclear retention of TAD mutants, or alternatively, forced cytosolic mis-localization of DBD mutants, would be capable of promoting the stabilization or function of nuclear and cytosolic EGFR respectively. We generated a series of TAD mutants lacking the nuclear export signal (NES) and DBD mutants lacking the nuclear localization signal (NLS) (Fig. 2F). Cytosolic EGFR levels were slightly elevated in DBD ΔNLS mutants, whereas nuclear EGFR levels were slightly elevated in TAD ΔNES mutants (Supplementary Fig. 5A, B). Conversely, cytosolic and nuclear EGFR levels were decreased in TAD ΔNES and DBD ΔNLS respectively. This effect was more subtle in H1299 cells expressing TAD ΔNES mutants, possibly due to cytosolic retention of some mutant p53.

These observations prompted us to test the effect of mis-localized mutants on EGFR signaling. Levels of phosphorylated EGFR (Y1068) in the cytosol of TAD ΔNES cells and in the nucleus of DBD ΔNLS cells decreased slightly (Supplementary Figs. 5C–H and 6A, B) in correlation with changes in EGFR levels in these cellular compartments (Supplementary Fig. 5A, B). DBD ΔNLS cells exhibited increased levels of cytosolic phosphorylated EGFR (Y1101) but not nuclear phosphorylated EGFR (Y1101), consistent with retention of EGFR in the cytosol due to impeded nuclear import. As expected, Cyclin D1 levels were also decreased in DBD ΔNLS cells (Fig. 2G and Supplementary Fig. 5I). Analysis of phosphorylated AKT revealed a striking increase in DBD ΔNLS mutant cells (Fig. 2H and Supplementary Fig. 5J). This increase in phosphorylated AKT correlated with DBD ΔNLS mutants gaining interaction with AKT (Fig. 2I). Furthermore, there was an increase in EGFR-AKT interaction in the cytosolic fraction of DBD ΔNLS cells. Conversely, there was neither an increase in nuclear phosphorylated EGFR (Y1101) (Supplementary Fig. 5E, G) nor a corresponding increase in Cyclin D1 levels (Fig. 2G and Supplementary Fig. 5I) in TAD ΔNES mutant cells. This striking observation suggests that while TAD ΔNES or DBD ΔNLS mutants were capable of stabilizing EGFR in the nucleus or cytosol respectively, these different forms of mutant p53 were not able to promote EGFR function equivalently in either intracellular compartment. In particular, TAD ΔNES mutants are able to stabilize EGFR in the nucleus, but unable to promote nuclear EGFR function.

### TAD mutants promote EGFR interaction with AKT while DBD mutants interfere with SHP1 binding to EGFR

**Fig. 3:**
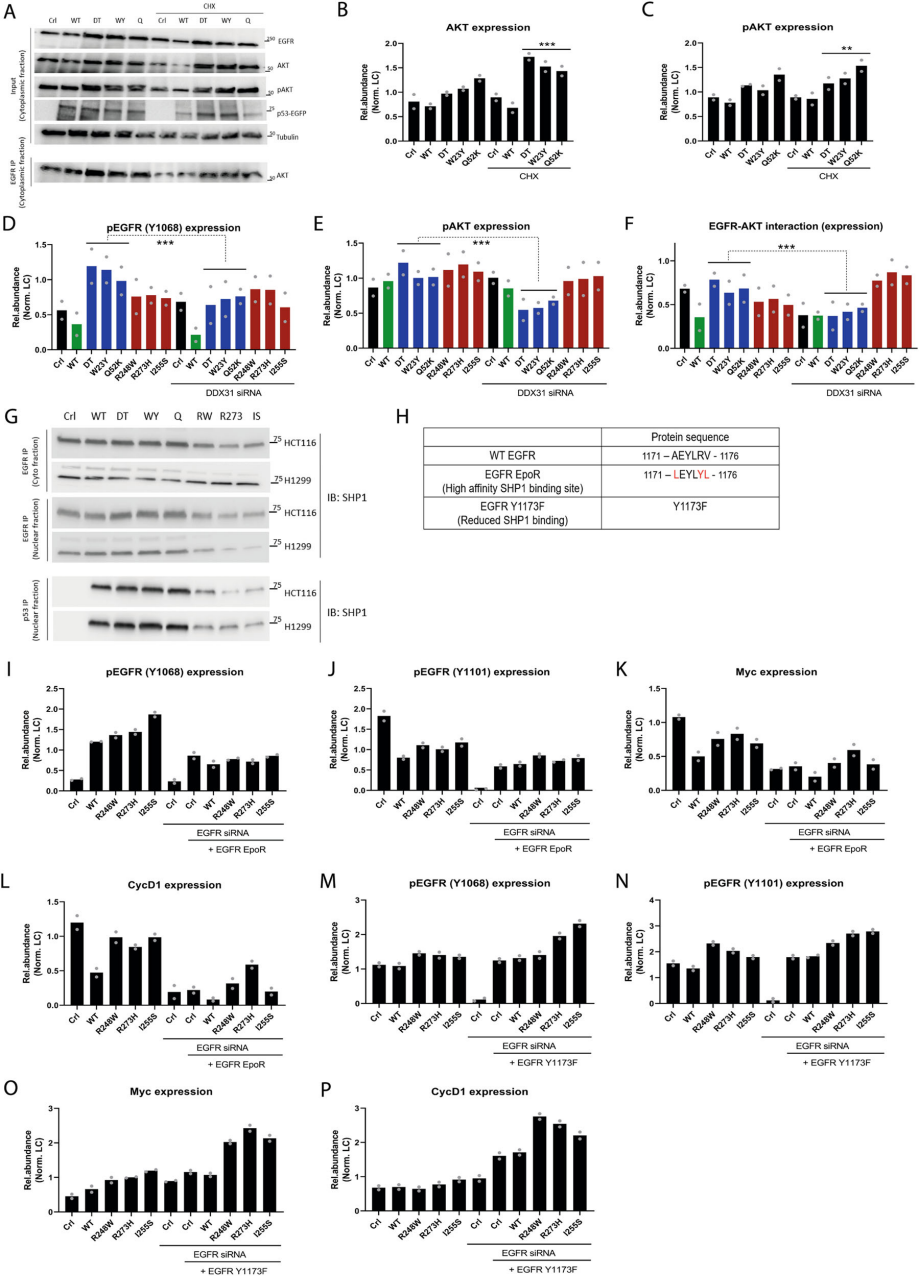
TAD mutants promote EGFR-AKT signaling while DBD mutants reduce EGFR-SHP1 interaction. **A** Protein levels of EGFR, AKT and pAKT in input cytosolic fractions from HCT116 cells expressing TAD mutants under untreated and cycloheximide-treated conditions. Protein levels of AKT pulled down with EGFR. *n* = 2. **B**, **C** Quantification of **B** AKT and **C** pAKT protein levels in HCT116 cells expressing TAD mutants under untreated and cycloheximide-treated conditions. Statistical tests performed on TAD (*n* = 3 independent mutants) versus TAD + CHX (*n* = 3 independent mutants). *n* = 2. **D**–**F** Quantification of cytosolic **D** pEGFR (Y1068), **E** pAKT, and **F** AKT (after EGFR pulldown) protein levels in HCT116 cells expressing WT and mutant p53 under untreated and DDX31 siRNA treated conditions. Statistical tests performed on TAD (*n* = 3 independent mutants) versus TAD + DDX31 siRNA (*n* = 3 independent mutants). *n* = 2. **G** Protein levels of SHP1 pulled down with EGFR or p53 in HCT116 and H1299 cells expressing WT and mutant p53. *n* = 2. **H** Table of EGFR mutants with contrasting affinities for SHP1 used in this study. **I**–**L** Quantification of **I**, **J** pEGFR, **K** c-Myc, and **L** Cyclin D1 protein levels in HCT116 cells expressing DBD mutants. Cells were treated with EGFR siRNA and expressed EGFR EpoR (high affinity for SHP1). *n* = 2. **M**–**P** Quantification of **M**, **N** p EGFR, **O** c-Myc, and **P** Cyclin D1 protein levels in HCT116 cells expressing DBD mutants. Cells were treated with EGFR siRNA and expressed EGFR Y1173F (reduced affinity for SHP1). *n* = 2. Two-tailed unpaired Student’s *t* test was performed. (*p < 0.05, **p < 0.01, ***p < 0.001, ****p < 0.0001). Source data are provided as a Source Data file.

How do TAD versus DBD mutants promote EGFR signaling in the cytosol and nucleus respectively? We first considered the mechanism of TAD mutant p53. Since AKT is a mutant p53 interactor enriched amongst TAD mutants (Fig. 1H), we determined the stability of AKT using a pulse-chase assay. AKT stability was enhanced in the presence of TAD mutants compared to control HCT116 and H1299 cells (Fig. 3A, B and Supplementary Fig. 7A, B). This in turn correlated with higher levels of phosphorylated AKT (Fig. 3C and Supplementary Fig. 7C).

In addition, the DEAD (Asp-Glu-Ala-Asp) box polypeptide 31 (DDX31) is known to interact with mutant p53 and EGFR^34^. Furthermore, cytoplasmic DDX31 can activate the EGFR-AKT signaling axis. We observed an increased interaction between TAD mutants and DDX31 (Supplementary Fig. 7D). There were also increased interactions between EGFR, AKT, and DDX31 in the presence of TAD mutants compared to DBD-expressing HCT116 and H1299 cells (Supplementary Fig. 7D). Significantly, these interactions with DDX31 were decreased in the presence of TAD ΔNES mutants but enhanced in the presence of DBD ΔNLS mutants, correlating with the increase in levels of phosphorylated AKT in DBD ΔNLS mutant cells (Fig. 2H and Supplementary Fig. 5J). We also confirmed the importance of TAD-EGFR-AKT-DDX31 interaction in regulating EGFR signaling by silencing DDX31. DDX31 silencing caused a significant decrease in levels of cytosolic phosphorylated EGFR (Y1068) and AKT, as well as reduced interaction between EGFR and AKT in TAD mutant HCT116 and H1299 cells but not DBD mutant ones (Fig. 3D–F and Supplementary Fig. 7E). Together, these data indicate that TAD mutants can promote EGFR-AKT signaling by enhancing interactions with known central axis protein partners such as DDX31.

Next, we considered the mechanism of DBD mutant forms of p53, which we observe to induce increased levels of phosphorylated EGFR. There are several phosphatases that are known to target EGFR such as PTP1B, PTPD1, SHP1, and SHP2^35^, raising the possibility that DBD mutant p53 might inhibit their effects. Indeed, we found that the interaction between EGFR and SHP1 was significantly reduced in the presence of DBD mutants (Fig. 3G and Supplementary Fig. 7F). The inability of TAD ΔNES mutants to elicit an increase in phosphorylated EGFR (Supplementary Figs. 5B, G, H and 6A, B) in the nucleus or Cyclin D1 expression (Fig. 2G and Supplementary Fig. 5I) was corroborated by lack of a decrease in interaction between EGFR and SHP1 (Supplementary Fig. 7G).

The region around phosphorylated EGFR (Y1173) has been identified as the binding site for SHP1^36^. Indeed, residues 1171 – 1176 of EGFR can be mutated to create a high affinity binding site for SHP1 (Fig. 3H), introducing a motif which was first identified in the erythropoietin receptor (EpoR). We therefore created a mutant form of EGFR (EGFR EpoR) incorporating the EpoR SHP1 binding motif. In the presence of EGFR EpoR, we observed an increased interaction between EGFR and SHP1 and a subsequent decrease in interaction between DBD mutants and EGFR in both HCT116 and H1299 cell lines (Supplementary Fig. 8E, F), which in turn, led to a reduction in phosphorylated EGFR, Cyclin D1 and c-Myc levels (Fig. 3I–L and Supplementary Fig. 8A–D). We also investigated the effect of the EGFR Y1173F mutant which is known to impair SHP1 binding (Fig. 3H). In the presence of mutant EGFR Y1173F, we observed increased interaction between EGFR and the DBD mutants (Supplementary Fig. 8K, L) and a corresponding increase in levels of phosphorylated EGFR, Cyclin D1, and c-Myc, particularly in HCT116 cells (Fig. 3M–P and Supplementary Fig. 8G–J).

Thus, our findings provide evidence that TAD mutants stimulate EGFR-AKT signaling by enhancing the interaction of EGFR with DDX31. By contrast, DBD mutants decrease the interaction of EGFR with the inhibitory phosphatase, SHP1.

**Fig. 4:**
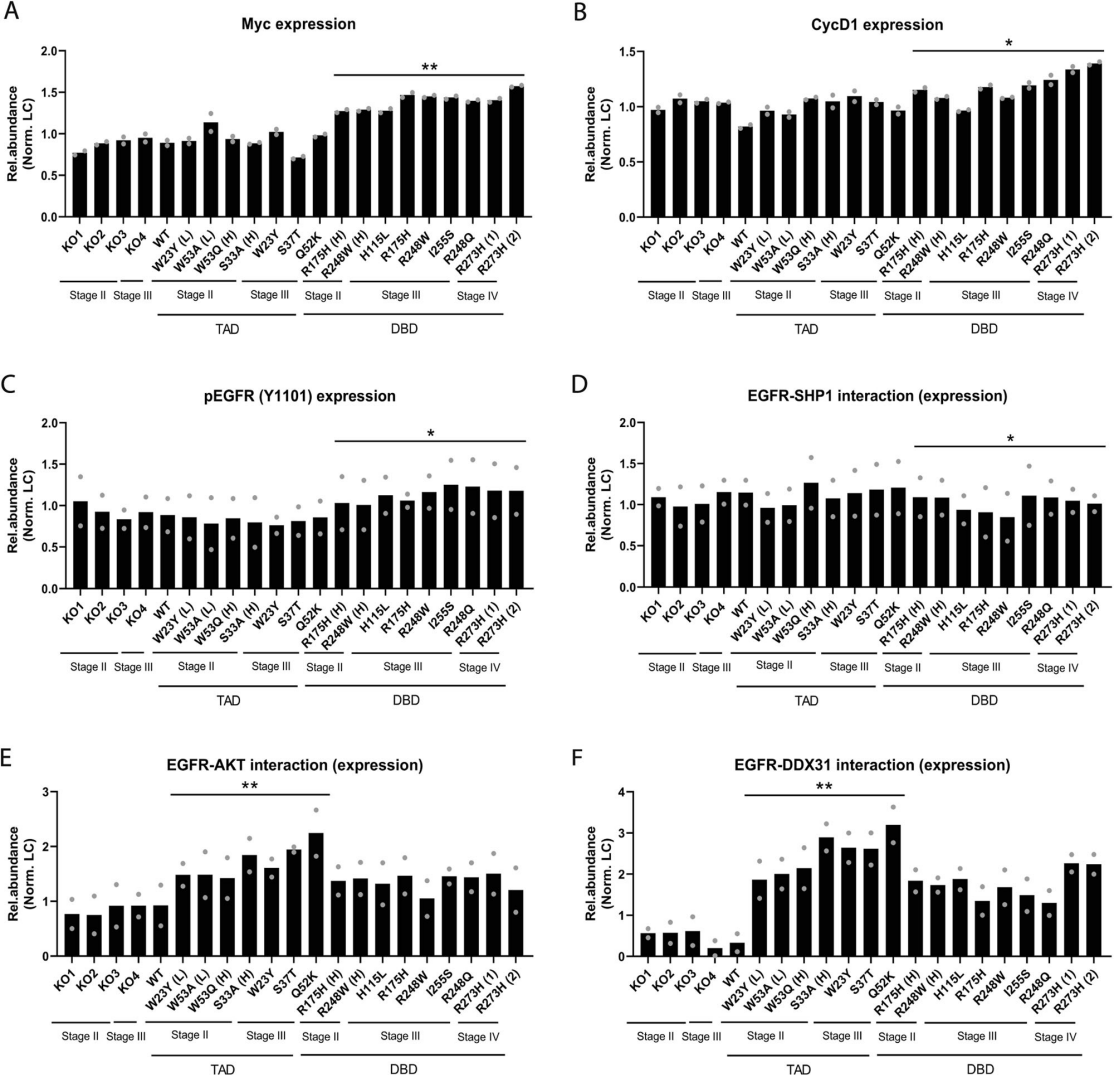
p53 TAD and DBD mutant CRC tumors recapitulate differential EGFR-dependent changes. **A**–**C** Quantification of **A** c-Myc, **B** Cyclin D1, and **C** nuclear pEGFR (Y1101) protein levels in p53 null and TAD and DBD mutant patient colorectal tumors. **D**–**F** Quantification of **D** nuclear SHP1 (after EGFR pulldown), **E** cytosolic AKT (after EGFR pulldown), and **F** cytosolic DDX31 (after EGFR pulldown) protein levels in p53 null and TAD and DBD mutant patient colorectal tumors. HCT116 cells expressing WT p53 as a control. All statistical tests performed on TAD (*n* = 7 independent patients) versus DBD (*n* = 9 independent patients). Two-tailed unpaired Student’s *t* test was performed. (*p < 0.05, **p < 0.01, ***p < 0.001, ****p < 0.0001). Source data are provided as a Source Data file.

We therefore tested the correlations between TAD and DBD mutants and modulation of EGFR activity in our CRC patient cohort. Pulldown assays using tumor tissue showed that the various p53 mutants in our panel interacted with EGFR, with TAD mutants demonstrating an increased association with cytosolic EGFR (Supplementary Fig. 9D). Levels of cytosolic EGFR and phosphorylated EGFR were elevated in TAD mutant tumors regardless of tumor stage (Supplementary Fig. 9A, B). Levels of Cyclin D1, c-Myc and nuclear phosphorylated EGFR (Y1101) were elevated in DBD mutant tumors in a stage-independent manner (Fig. 4A–C and Supplementary Fig. 9C, D) and EGFR interaction with SHP1 was reduced in DBD mutant tumors (Fig. 4D and Supplementary Fig. 9E), corroborating our findings in HCT116 and H1299 cells. There was increased interaction between EGFR and AKT in TAD mutant CRC tumors (Fig. 4E and Supplementary Fig. 9D) and this correlated with higher levels of phosphorylated AKT (Supplementary Fig. 9F, G). Only TAD mutants were capable of interaction with AKT (Supplementary Fig. 9D) and there was increased association between EGFR and DDX31 in the presence of TAD mutants (Fig. 4F and Supplementary Fig. 9D). These observations corroborate in tumor tissues our findings that TAD mutants can promote EGFR-AKT signaling through direct interactions that likely stabilize this protein signaling complex.

Together, our findings indicate that both TAD and DBD mutants selectively regulate EGFR activity via separate domain-specific mechanisms. These mechanisms are primarily dependent on specific protein interactions ‘gained’ by mutant p53 that stabilize or disrupt signaling complexes. TAD mutants facilitate the interaction between EGFR and downstream kinases such as AKT, while DBD mutants reduce binding of the inhibitory phosphatase, SHP1 to active, phosphorylated EGFR. These molecular changes were also observed in CRC tumors, suggestive of their potential significance in tumor development, as well as the clinical implications of our findings.

### TAD and DBD mutants interact with EGFR at unique sites

**Fig. 5:**
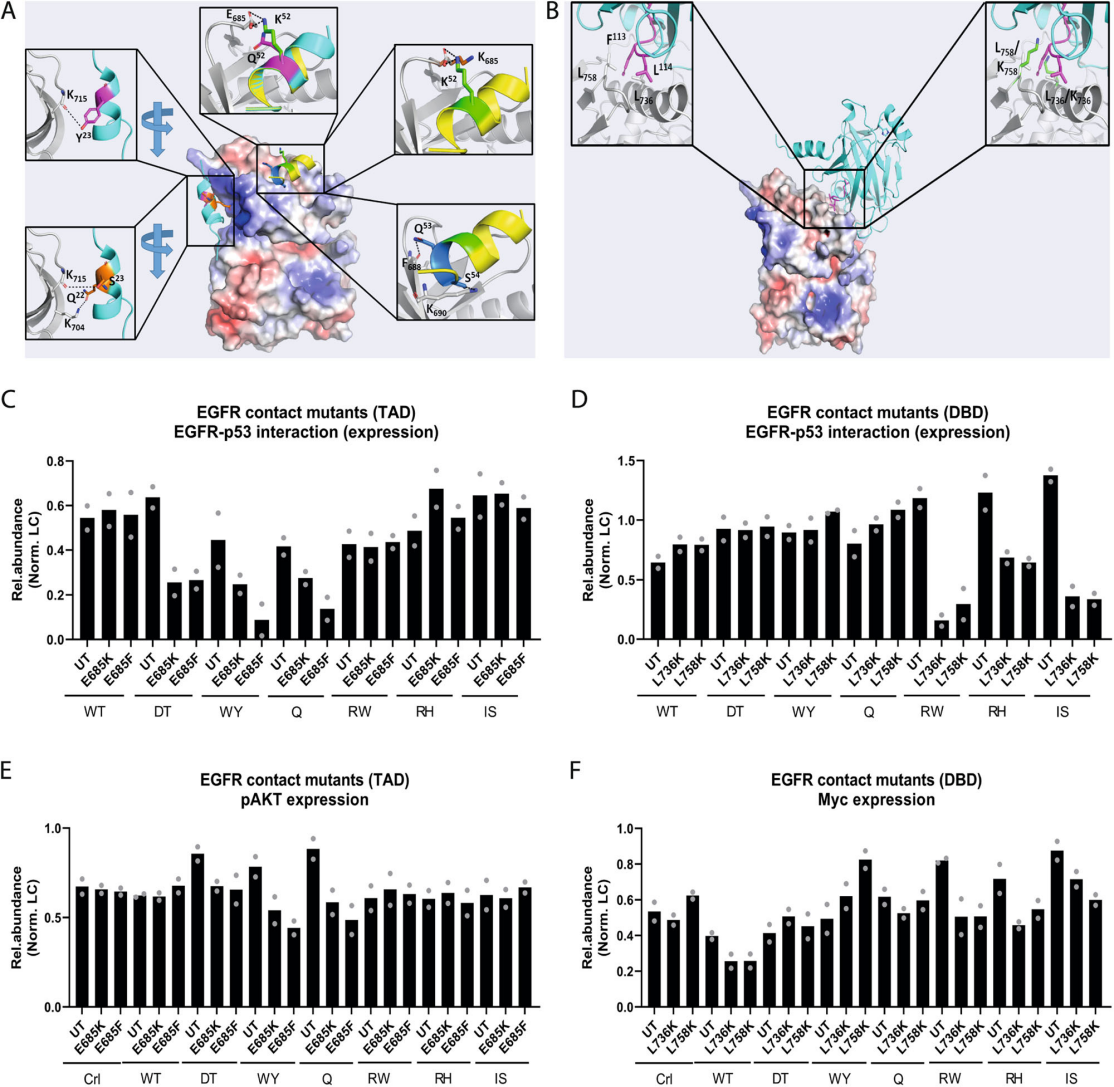
TAD and DBD mutants interact with EGFR at specific sites. **A** Structure of the modeled EGFR-p53TAD mutant complexes. EGFR is shown with its surface colored by the electrostatic potential (with red to blue colors corresponding to potentials of −5 kcal/mol to + 5 kcal/mol) and the p53 TAD peptides shown as cartoon (TAD1: Cyan, TAD2: yellow). Top middle: zoom in view of the EGFR-p53TAD2 complex, with EGFR and p53TAD2 shown as cartoon, with residues E685 from EGFR and K52/Q52 from TAD2 shown as sticks; the likely salt bridge between E685 and K52 is highlighted in dashed lines (black). Top right: zoom in view of the EGFR-p53TAD2 complex, with EGFR and p53TAD2 shown as cartoon and the suggested mutation E685K in EGFR and Q52K in TAD2 shown as sticks. Bottom right: view of the EGFR-p53TAD complex highlighting the interactions (black dashed lines) between residue F688 from EGFR with residue Q53 from TAD2 and K690 from EGFR with residue S54 from TAD2. Top left: zoom in view of the EGFR-p53TAD1 complex with residue K715 from EGFR and W23Y from Tad1 shown as sticks; the likely hydrogen bond interaction between K715-Y23 is highlighted in dashed lines (black). Bottom left: zoom in view of the EGFR-p53TAD1 complex highlighting the interactions (back dashed lines) between residue K704 from EGFR and residue Q22 from TAD1 and K715 from EGFR and residue S23 from TAD1. **B** Structure of EGFR-p53DBDR175H complex. EGFR is shown with its surface colored according to the electrostatic potential (with red to blue color corresponding to −5kcal/mol to + 5 kcal/mol) and the p53DBD-R175H shown as cartoon (Cyan) with the solvent exposed hydrophobic patch highlighted in magenta. Top left: zoom in view of EGFR-p53 DBD-R175H shown as cartoon and residues F113, L114 from p53 DBD and L736, L758 from EGFR shown as sticks. Top right: zoom in view of the mutant EGFR-p53DBD-R175H complex, highlighting the disruption of hydrophobic interactions due to L736K and L758K mutations. **C**, **D** Quantification of EGFR (after p53 pulldown) protein levels in HCT116 cells expressing (C) EGFR mutants that disrupt TAD binding (E685K, E685F) and **D** mutants that disrupt DBD binding (L736K, L758K). *n* = 2. **E**, **F** Quantification of pAKT and c-Myc protein levels in HCT116 cells expressing **E** EGFR mutants that disrupt TAD binding (E685K, E685F) and **F** mutants that disrupt DBD binding (L736K, L758K). *n* = 2. Source data are provided as a Source Data file.

The N-terminal region of p53 is generally disordered with two main regions of localized order – TAD1 and TAD2 (Fig. 1A) – that are known to adopt a helical conformation upon target binding^10^. This region of p53 has been reported to interact with the N-terminal domain of the focal adhesion kinase (FAK)^37^. Given the homology between the N-terminal domains of FAK and EGFR, we modeled a p53TAD-EGFR complex on this structural basis. We focused on the structured TAD domains of p53 (residues: 14-26 and 46-56) and modeled alpha helical conformations for several relevant TAD mutants – Q52K, W23Y, W23S, and WF53/54QS (Fig. 5A). We defined the interfacial sites of binding to EGFR as the solvent exposed region of the N-lobe of the EGFR kinase domain (residues: 693, 694, 703 – 706, 713 – 717, and 756 – 764). In our modeled p53-EGFR complex, we found that, for example, the mutated TAD2 domain could bind the N-lobe of EGFR and that the mutant residue K(Lys)52 interacted with E(Glu)685 of EGFR (Fig. 5A). The resultant formation of a salt bridge between K(Lys)52 of the mutant p53 Q52K and E(Glu)685 of EGFR would enable tighter binding of the two proteins. Subsequent modeling indicated that replacement of E(Glu)685 with a lysine residue is likely to reduce or abolish interaction between p53 Q52K and EGFR due to charge-charge repulsions. We also considered the effect of posttranslational modifications of TAD mutants on their ability to bind better to EGFR. For example, W23Y is predicted to have an increased affinity for phosphorylated residues, which might further enhance interaction with EGFR due to interfaces between the negatively charged phosphate and the positively charged portion of EGFR (Supplementary Fig. 10A).

To investigate the structural basis for the DBD-EGFR interaction, we examined molecular dynamics simulations of the DBD mutant R175H. We found that an FXXXF hydrophobic motif (109 – FRLGFL – 114) in R175H, which is buried in the simulations of WT p53, is exposed in R175H. We then explored the surface of EGFR and identified a potential hydrophobic cleft between the S60-L68 helix and C89-V94 turn and postulated that this cleft may be a docking site for the FXXXF motif in R175H. To model the EGFR-DBD mutant (R175H) protein complex, we defined the binding sites of EGFR as the alphaC helix (residues: 728–744), beta3-alphaC helix loop (residues: 723 – 727), beta4 strand (residues: 752–757), beta4-beta5 loop (residues: 757–762) and the corresponding sites of p53 R175H as the exposed FXXXF motif (residues: 109–114). From the docking poses generated, we found that the solvent-exposed hydrophobic region of R175H indeed bound the PIF-like pocket of EGFR, with residues F113 and L114 buried into the hydrophobic pocket (Fig. 5B). Subsequent modeling indicated that replacement of L(Leu)736 or L(Leu)758 with lysine residues will reduce or abolish interaction between p53 R175H and EGFR due to disruption of hydrophobic interactions. This was also the case for other common DBD mutants such as R248W and I255S (Supplementary Fig. 10B, C).

To validate these structural models, we generated various EGFR contact mutants and expressed them in HCT116 cells. EGFR point mutants E685K and E685F demonstrated reduced interaction with TAD mutants (Fig. 5C and Supplementary Fig. 10D) while L736K and L758K exhibited reduced interactions with DBD mutants (Fig. 5D and Supplementary Fig. 10D). Correspondingly, reduced interactions between TAD and EGFR contact mutants (E685K and E685F) resulted in a decrease in levels of phosphorylated AKT (Fig. 5E and Supplementary Fig. 10E). Meanwhile, reduced interactions between DBD and EGFR contact mutants (L736K and L758K) led to decreased levels of c-Myc (Fig. 5F and Supplementary Fig. 10E). These findings demonstrate that domain-specific *TP53* mutants interact with EGFR at unique sites, which may contribute to EGFR stability, and increased cytosolic and nuclear signaling in TAD and DBD mutant cells respectively.

### *TP53* TAD mutations confer resistance to EGFR inhibition

Resistance to EGFR inhibition presents a significant challenge in cancer therapeutics. Hence, the ability to predict efficacy under conditions where multiple oncogenic signaling pathways are active is an important goal. Our results already reveal that p53 DBD mutant cells were more sensitive to EGFR inhibition (Supplementary Fig. 3E, I) using Erlotinib. We further investigated the effect of Erlotinib on the distinct modes of EGFR activation that are present in TAD and DBD mutant cells.

**Fig. 6:**
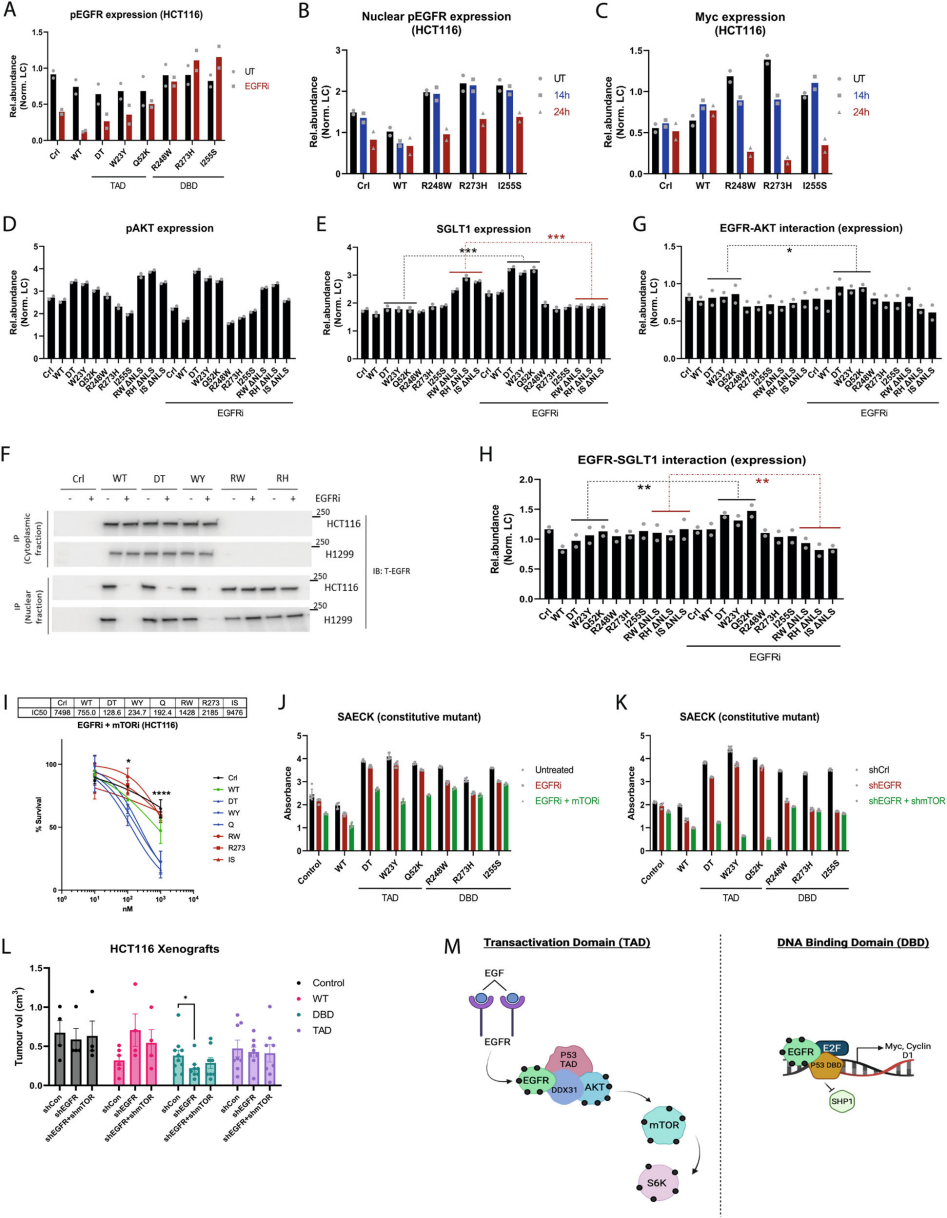
Presence of TAD mutants confer resistance to EGFR inhibition. **A** Quantification of pEGFR (pan) protein levels in HCT116 cells expressing WT and mutant p53 treated with EGFR inhibitor. *n* = 2. **B**, **C** Quantification of **B** nuclear pEGFR (pan) and **C** whole-cell c-Myc protein levels in HCT116 cells expressing WT and mutant p53 treated with EGFR inhibitor. *n* = 2. **D**, **E** Quantification of **D** pAKT and **E** SGLT1 protein levels in HCT116 cells expressing WT and mutant p53 and treated with EGFRi. *n* = 2. **F** Protein levels of cytosolic and nuclear EGFR pulled down with p53 in HCT116 and H1299 cells expressing WT and mutant p53. *n* = 2. **G**, **H** Quantification of **G** AKT (after EGFR pulldown) and **H** SGLT1 (after EGFR pulldown) protein levels in HCT116 cells expressing WT and mutant p53 and treated with EGFRi. *n* = 2. **I** Dose response curves of HCT116 cells expressing WT and mutant p53 treated with EGFR inhibitor in combination with mTORi. *n* = 3 independent experiments. **J**, **K** Quantification of crystal violet intensity from colony formation assays using SAECK cells constitutively expressing WT and mutant p53. Cells were treated with **J** EGFRi or EGFRi+mTORi and **K** inducible expression of shControl or shEGFR and/or shmTOR *n* = 4 independent replicates. Error bars denote Mean ± SEM. *p* < 0.0001 for DBD untreated vs DBD EGFRi/DBD shEGFR and TAD untreated vs TAD EGFRi+mTORi/shEGFR+shmTOR (*n* = 3 mutants each). **L** Quantification of tumor volume of xenograft tumors derived from HCT116 cells constitutively expressing WT and mutant p53 with inducible knockdown of EGFR and mTOR. *n* = 2–5 mice and *n* = 4-10 tumors per mutant per shRNA condition. Individual data points denote individual tumors. Statistical tests performed on TAD or DBD shControl versus shEGFR or shEGFR+shmTOR. Two-tailed unpaired Student’s *t* test was performed. (*p < 0.05, **p < 0.01, ***p < 0.001, ****p < 0.0001). **M** Model of TAD and DBD mutant p53 modulation of cytoplasmic and nuclear EGFR functions respectively. Created with BioRender.com. Source data are provided as a Source Data file.

DBD mutant HCT116 and H1299 cells maintained high levels of phosphorylated EGFR after 14 hours of treatment with Erlotinib (Fig. 6A and Supplementary Fig. 11A, B). Levels of phosphorylated EGFR only decreased significantly when DBD mutant cells were treated for at least 24 h with Erlotinib (Fig. 6B and Supplementary Fig. 11C, G) and this correlated with marked reduction in c-Myc levels in DBD mutant cells as compared to control or cells expressing WT p53 (Fig. 6C and Supplementary Fig. 11D–G). Meanwhile, despite a decrease in levels of phosphorylated EGFR in the presence of TAD mutants following Erlotinib treatment, these cells exhibited reduced sensitivity to EGFR inhibition as compared to DBD mutant cells (Supplementary Fig. 3E, I). We examined levels of phosphorylated AKT and S6K and found that levels were maintained in TAD mutant cells (Fig. 6D and Supplementary Fig. 11B, H, J). SGLT1 is known to interact with EGFR independently of EGFR tyrosine kinase activity and EGFR inhibition does not perturb this interaction^38^. This allows for maintenance of high levels of intracellular glucose and confers a survival advantage. SGLT1 levels were elevated in TAD mutant cells and levels were generally maintained or further increased in response to Erlotinib treatment (Fig. 6E and Supplementary Fig. 11I, J). As mutant p53 interacts with and stabilizes active EGFR, we did pulldowns to determine if this interaction was maintained in the presence of Erlotinib. TAD mutants interacted with both active and inactive EGFR in the cytosolic fraction (Fig. 6F), potentially supporting continued interaction between inactive EGFR and downstream factors such as AKT and facilitating signaling through them. DBD mutants maintained their interaction with inactive EGFR only in the nucleus which correlated with sustained levels of phosphorylated EGFR after 14 h of Erlotinib treatment. Interestingly, the reduction in interaction between DBD mutants and EGFR contact mutants (L736K and L758K) conferred even greater sensitivity to EGFR inhibition (Supplementary Fig. 11K, L). Furthermore, DBD mutant cells generally remained more sensitive to EGFR inhibition than TAD mutant cells, corroborating our earlier findings (Supplementary Fig. 3E, I) and suggesting that the increased sensitivity of DBD mutant cells to EGFR inhibition may be related to their greater reliance on nuclear stabilization and activity of EGFR. In contrast, to TAD mutants may confer some degree of resistance to EGFR inhibition by promoting and stabilizing interactions between EGFR and AKT or SGLT1.

We have demonstrated that the forced mis-localization of DBD mutants (DBD ΔNLS) to the cytosol led to recapitulation of TAD-specific molecular characteristics such as increased levels of phosphorylated AKT (Fig. 2H and Supplementary Fig. 5J) and increased EGFR-AKT interaction (Fig. 2I). However, we found that DBD ΔNLS mutant cells were no more resistant to Erlotinib than DBD mutant cells (Supplementary Fig. 12A–D). We observed that following Erlotinib treatment, levels of phosphorylated AKT and interaction between inactive EGFR and AKT were maintained in DBD ΔNLS mutant cells (Fig. 6D, G and Supplementary Fig. 11H and 12E, F). We next investigated SGLT1 levels in Erlotinib-treated DBD ΔNLS mutant cells and found that, unlike in TAD mutant cells, there was no increase in SGLT1 levels (Fig. 6E and Supplementary Fig. 11I). The lack of increase in SGLT1 levels in DBD ΔNLS mutant cells also correlated with reduced interaction between inactive EGFR and SGLT1 (Fig. 6H and Supplementary Fig. 12E, G). Meanwhile, the lack of an increased sensitivity of TAD ΔNES mutant cells to EGFR inhibition might be attributed to our observations that nuclear retention of these mutants did not correspondingly affect nuclear phosphorylated EGFR (Y1101) (Supplementary Fig. 5G) or Cyclin D1 levels (Fig. 2G and Supplementary Fig. 5I). These findings highlight that interaction between EGFR and SGLT1 is important in conferring relative resistance to Erlotinib in TAD mutant cells compared to DBD mutant ones.

Given that the interaction between DBD mutants and EGFR reduced SHP1 binding and dephosphorylation of EGFR, we sought to determine whether modulating SHP1 interaction could directly impact sensitivity of DBD mutant cells to EGFR inhibition. To this end, we depleted WT EGFR in DBD mutant HCT116 cells, and expressed the EGFR mutants EGFR EpoR (increased SHP1 binding) or EGFR Y1173F (reduced SHP1 binding). In line with our earlier observations, the presence of EGFR EpoR further increased the sensitivity of DBD mutant cells to Erlotinib while the EGFR YF mutant reduced sensitivity (Supplementary Fig. 12H–K) and was accompanied by changes in EGFR-SHP1-DBD mutant interactions (Supplementary Fig. 8E, F, K, L).

Next, we determined the sensitivity of TAD mutant HCT116 and H1299 cells to combinations of Erlotinib with AKT or PI3K or mTOR inhibitors. TAD mutant cells were indeed more sensitive to all three of these dual drug combinations (Fig. 6I and Supplementary Fig. 12L–P). We also investigated the impact of perturbing EGFR and mTOR activity on colony formation ability of TAD versus DBD mutant cells. For this, we generated HCT116 or human small airway epithelial cells (SAECK) constitutively expressing TAD and DBD mutants followed by drug inhibitor treatment or stable, inducible shRNA knockdown of EGFR and mTOR (Supplementary Fig. 12S). We found that DBD mutant HCT116 and SAECK cells were more sensitive to Erlotinib and formed smaller colonies compared to TAD mutants (Fig. 6J and Supplementary Fig. 12Q). This difference was more significant with stable knockdown of EGFR, especially in SAECK cells (Fig. 6K and Supplementary Fig. 12R). Meanwhile, treating TAD mutant HCT116 and SAECK cells with both Erlotinib and mTOR inhibitor effectively reduced colony formation ability and again, there was a more significant effect in the presence of EGFR and mTOR double knockdown (Fig. 6J, K and Supplementary Fig. 12Q, R).

We also conducted in vivo xenograft experiments using HCT116 constitutive TAD and DBD mutant cells with inducible EGFR and/or mTOR knockdown. We observed that, similar to their behavior in colony-forming assays, DBD mutant cells were significantly more sensitive to EGFR knockdown and formed smaller tumors (Fig. 6L and Supplementary Fig. 12S). Combined EGFR and mTOR knockdown also tended to reduce the size of resultant TAD mutant tumors, but this effect was more variable, and hence, did not reach statistical significance compared to control (Fig. 6L).

Overall, our findings highlight that *TP53* mutations may confer increased sensitivity to EGFR inhibition both in vitro and in vivo, particularly in DBD mutant tumors, and that this effect arises from the unique mechanisms by which these mutants can modulate EGFR activity and function.

## Discussion

While differences in the tumor-suppressive and pro-carcinogenic properties of different p53 mutant proteins are recognized, our work provides a first example to highlight that distinct molecular mechanisms underlie the effects of domain-specific mutants, and can be used to differentiate cancers carrying them. We have focused on clinically relevant mutations within two critical domains of p53. Our findings reveal previously unknown, tissue-independent molecular mechanisms by which TAD and DBD mutants activate pro-oncogenic EGFR signaling. We propose a model (Fig. 6M) in which - through binding and stabilizing EGFR in different compartments - TAD and DBD mutants enhance pro-carcinogenic EGFR signaling via different mechanisms. TAD mutants promote canonical EGFR signaling in the cytosol and enhance the interaction between EGFR and AKT via DDX31. By contrast, DBD mutants maintain levels of phosphorylated EGFR in the nucleus by disrupting SHP1 binding and promoting EGFR interaction with transcription factors such as E2F1, enabling EGFR to regulate c-Myc and Cyclin D1 levels. Our findings have several important implications.

Our findings indicate that p53 DBD mutants alter EGFR signaling in the nuclear compartment. The non-canonical nuclear localization and function of EGFR have been studied extensively, and greater clarity is emerging about their impact on tumor development^39^. Nuclear EGFR localization has been associated with highly proliferative cells, therapy resistance and worse prognosis in multiple cancers^31^. Apart from functioning as a co-transcription factor, nuclear EGFR has been implicated in DNA replication through stabilization of PCNA, and in DNA repair through activation of DNA-PKs and ERCC1^40,41^. Given our findings that DBD mutants stabilize EGFR in the nucleus and that these cells exhibit increased levels of genomic instability, further investigation of EGFR modulation of DNA repair in the presence of domain-specific p53 mutants might be informative.

By contrast, our findings show that TAD mutants can stabilize cytosolic EGFR and promote downstream AKT signaling. It has been shown that following EGFR endocytosis, EGFR-AKT signaling can occur within early and late endosomes prior to lysosomal degradation and/or recycling of EGFR to the cell surface^42^. Early endosomes expressing the Rab5 effector, APPL1^43^, EEA1^44^, or phosphoinositides^45^ can activate AKT activity. Increased AKT activity can in turn modulate EGFR endocytic trafficking by activating PIKfyve (FYVE-containing phosphatidylinositol 3-phosphate 5-kinase) which promotes lysosomal degradation as a feedback mechanism^46,47^. These findings raise the possibility that TADs may facilitate EGFR-AKT signaling – either via stabilization of EGFR within early endosomes or inhibiting feedback lysosomal degradation – which warrants future investigation. The significance of DDX31 in maintaining interaction between TAD mutants and the EGFR-AKT complex and promotion of cytosolic EGFR signaling also highlights the therapeutic potential of targeting novel protein complexes formed by mutant p53. Interactions between nuclear DDX31, mutant p53, and nucleolar proteins have been reported to modulate EGFR signaling in bladder cancer and increased cytosolic expression of DDX31 and mutant p53 have been implicated in poorer prognosis^34^.

Collectively, our analyses in vitro concerning the mechanisms of TAD and DBD mutants indicate that they may exert pro-carcinogenic effects by activating EGFR signaling via distinct molecular interactions. This is in addition to reported classical GOF mechanisms wherein DBD ‘hotspot’ mutants can transactivate various gene targets through interaction with new transcription factors. We have further demonstrated that the distinct modes of EGFR activation by TAD and DBD mutants are relevant in human CRC in vivo. Our xenograft experiments further support the differential therapeutic vulnerabilities of at least DBD mutant cells, further highlighting their contribution to tumor development in vivo. Moving forward, given the diverse repertoire of understudied mutations occurring in other p53 domains, we need a clearer understanding of the contexts in which domain-specific mutants might exhibit similar properties and what these properties are. *TP53* germline mutations of unknown clinical significance are also being increasingly identified in individuals with familial predisposition syndromes like Li-Fraumeni syndrome (LFS)^48,49^ and somatic variants have been reported in morphologically normal, pre-cancerous tissue^50,51^. Deriving guiding principles for clinical management from domain-specific molecular characteristics of these mutants will be very useful.

Therapeutic targeting of the non-canonical functions of EGFR has also emerged as a strategy for overcoming different modes of drug resistance^19,52,53^. Our findings provide several insights. First, based on our findings with the EGFR EpoR mutant (which engages SHP1 with high affinity) and the EGFR Y1173F mutant (which reduces SHP1 binding), small molecules disrupting the DBD-EGFR-SHP1 axis may have therapeutic merit. Second, activation of downstream pathways such as AKT, PI3K, or MAPK has been implicated in EGFR inhibitor resistance, and the relative efficacy of dual inhibition of EGFR and downstream targets has been reported^54–56^. Our observations that TAD mutant cells are uniquely dependent on PI3K/AKT/mTOR signaling and that combining EGFR inhibition with relevant inhibitors targeting these pathways significantly impacted viability in vitro, further support the utility of combination therapy. Third, our findings that TAD mutants can enhance interaction between EGFR and AKT via DDX31 further highlight how domain-specific properties of mutant p53 may influence the prediction of therapeutic efficacy, patient stratification and the development of drugs that disrupt the TAD-EGFR-DDX31-AKT axis. Finally, given the frequent co-occurrence of *TP53* mutations and EGFR amplification in different tumor types, our findings suggest that targeting TAD- and DBD-specific modulation of EGFR activity could circumvent current challenges in directly targeting p53, or overcome resistance to EGFR inhibitors. In NSCLC, EGFR inhibitor resistance is commonly attributed to acquired *EGFR* T790M mutations or c-MET amplification. More recently, it has been recognized that *TP53* exon 8 mutations, which encompasses the DBD region, may influence NSCLC responsiveness to EGFR inhibition in patients harboring *EGFR* mutations^57,58^. The Lung Cancer Mutation Consortium (LCMC) has also reported that *TP53* mutations are the most commonly occurring event together with *EGFR* mutations^59^. However, mis-sense mutations in the TAD were categorized as ‘non-disruptive’^60^. Given our findings that DBD and TAD mutants differentially modulate EGFR, their impact on EGFR inhibitor treatment in EGFR mutant tumors warrants further investigation.

Taken together, the mechanistic insights reported here further underscore that *TP53* mutations in cancer should not be considered as a singular class. Domain-specific, tissue-independent properties of mutant p53 can potentially serve as indicators of clinical outcomes, and also inform the rational design of more selective and efficacious therapies.

## Methods

### Patient recruitment

CRC patients undergoing surgery were recruited from the National University Hospital (NUH). Information on *TP53* mutational status and tumor Stage (including risk of metastatic or recurrent diseases for Stage II patients) of our patient cohort are provided in Supplementary Data 1. Tumor and adjacent normal colorectal tissues were collected post-operatively. Tumor dissection was done by a pathologist and the tumor core was defined spatially as the center of the tumor specimen. The invasive front was defined as the tumor region that was either facing the submucosal or subserosal fronts. The 2 cm and 5 cm samples are taken in relation to the general position of the tumor. *TP53* mutational status was determined by genetic sequencing of the different tumor sections collected for each patient. Tumors designated as p53 null/knockout (KO) were verified by genetic sequencing for presence of truncating mutations and by western blot analysis. This study was approved by the relevant Institutional Review Boards (IRBs) in Singapore (DSRB Ref: 2018/01168), and informed consent was obtained from all research participants.

### Patient clinical datasets

All patient clinical data was mined using cBioportal. P53 domains were classified as follows: TAD: amino acid residue 1–61; PRD: 61–94; DBD: 94–292, OD: 325–356; and CTD: 356–393.

### Cell lines

HCT116 (ATCC CCL-247), H1299 (p53 null) (ATCC CRL-5803), and MCF7 (ATCC HTB-22) cells were cultured according to ATCC guidelines. SAECK cells generated by transducing SAEC parental cells (ATCC CRL-4050) with retroviruses generated from pBABE-puro SV40 LT (Addgene #13970), pBABE-hygro-hTERT (Addgene #1773), and pBABE puro K-Ras V12 (Addgene #9052) plasmids, selected with puromycin and hygromycin B and maintained in epithelial cell culture media^61^. In order to generate stable, doxycycline-inducible lines expressing WT and TAD and DBD mutant p53, HCT116 cells were engineered using CRISPR/Cas9 technology and knockout of p53 was verified using western blotting and sequencing. MCF7 cells stably expressing p53 shRNA were used. HCT116, H1299, MCF7, and SAECK cells were then lentivirally transduced with the relevant p53-EGFP (C-terminal tag) constructs. High to medium EGFP-expressing cells were FACS sorted. Likewise, HCT116 and SAECK cells constitutively expressing WT and TAD and DBD mutant p53 were lentivirally transduced with the relevant p53-EGFP (C-terminal tag), EGFR and/or mTOR shRNA constructs and FACS sorted and selected with puromycin respectively. All cell lines were routinely tested for mycoplasma.

### Xenografts

Animal experimentation was performed in accordance with the A*STAR Biological Resource Center guidelines (IACUC Protocol #211598). Live cells were resuspended in 50% Matrigel (Corning Basement Membrane Matrix) and 50% cell culture media. 1 million cells were injected per site, subcutaneously, into age-matched male or female NOD-*scid* IL2Rgamma^null^ (NSG) mice (InVivos). Water supply was supplemented with 2 mg/ml doxycycline (Sigma, D9891). Mice were sacrificed at 4 weeks latency (with individual tumors not exceeding 1.5cm^3^ in volume), All tumors were harvested at the same time.

### Immunofluorescence

Tissues were fixed in 4% formaldehyde in PBS overnight at room temperature before storage in 70% ethanol at −20 °C until embedding. Tissues were embedded in wax and sectioned at 3 µm thickness onto glass slides. Sections were dewaxed with xylene, rehydrated with an ethanol series followed by blocking in 5% goat serum in 0.1% Triton X-PBS for 1 h followed by overnight incubation with primary antibodies (anti-p53 (DO-1, Santa Cruz Biotechnology, SC-126, 1:100), anti-γH2AX (Millipore, #05-636, 1:2000), and anti-Ki67 (Invitrogen, #MAS-14520, 1:300)). Slides were washed thrice in 0.1% Triton X-PBS followed by incubation with secondary Alexa fluorophore-conjugated antibodies (Molecular Probes, all 1:500) for 1 h at room temperature. Nuclei were counterstained with Hoechst 33342 for 10 min and sections mounted in Vectashield Antifade mounting solution (Vector Laboratories).

Cells were seeded on glass-bottom chambered coverslips (ibidi) and fixed in 4% paraformaldehyde for 15 min at room temperature. Cells were permeabilized with 0.3% Triton X-PBS for 15 min before blocking with 5% BSA in 0.1% Triton X-PBS for 1 h. Cells were incubated with primary (overnight) and secondary Alexa fluorophore-conjugated antibodies as above. Nuclei were counterstained with Hoechst 33342. Slides were visualized with a confocal microscope (Zeiss, Leica) using ×20, ×40 or ×60 objective lenses.

### Image acquisition and analysis

For tissue sections, at least 10 fields of view were acquired at ×20 (Leica). For cells seeded on coverslips, at least 20 fields of view were acquired at ×40 (Zeiss). Images were analyzed using Image J and the number of cells staining positive for the protein of interest was quantified and expressed as a percentage of the total number of cells present in a given field of view. Histograms were plotted using GraphPad Prism and reflect mean ± SEM from at least two independent experiments.

### P53 reporter assay

ARN8 cells stabling expressing the p53 reporter RGCΔFos-LacZ^62–64^ were transfected with various p53 TAD and DBD mutants using Lipofectamine 3000 (Invitrogen). After 24 h, cells were lysed and β-galactosidase activity was determined using the substrate CPRG. Absorbance of the enzymatic product chlorophenol red was measured at 595 nm using an EnVision plate reader (PerkinElmer).

### Subcellular fractionation

Cell pellets were lysed with cytoplasmic extraction buffer (50 mM Tris HCl pH7.5, 100 mM NaCl, 0.5% NP-40, 1 mM EDTA, 1 mM DTT, 0.2 mM sodium fluoride, 0.2 mM sodium vanadate, protease inhibitor cocktail (Roche)) and incubated on ice for 30 min with gentle vortexing every 5 min before centrifuging at 500×*g* for 5 min at 4 °C. Supernatant was transferred to a fresh tube as the cytoplasmic fraction. The pellet was then resuspended in nuclear extraction buffer (50 mM Tris HCl pH7.5, 300 mM NaCl, 5 mM CaCl2, 0.2 mM sodium fluoride, 0.2 mM sodium vanadate, and protease inhibitor cocktail (Roche)) and incubated on ice for 20 min with vortexing every 5 min before centrifuging at 14,000 × *g* for 20 min at 4 °C. The supernatant was harvested as the nuclear soluble fraction.

50 mg of frozen, ground colonic tissue was homogenized in 200 μL of buffer 1 (10 mM HEPES-potassium hydroxide pH7.9, 10 mM potassium chloride, 2 mM magnesium chloride, 0.5 mM DTT, 0.1 mM EDTA, 0.1 mM EGTA, 0.2 mM sodium fluoride, and protease inhibitor cocktail (Roche)). Samples were incubated on ice for 15 min before 15 μL of buffer 2 (10% NP-40) was added. Samples were vortexed for 30 s and centrifuged at 14,000 × *g* for 5 min at 4 °C to pellet nuclei. The supernatant was collected as the cytoplasmic fraction. The pellet was resuspended in 80 μL of buffer 3 (50 mM HEPES-potassium hydroxide pH7.9, 50 mM potassium chloride, 300 mM sodium chloride, 1 mM DTT, 0.1 mM EDTA, 10% glycerol, 0.2 mM sodium orthovanadate, protease inhibitor cocktail (Roche)) and incubated for 20 min on ice with vortexing every 5 min. Samples were then centrifuged at 14,000 × *g* for 5 min at 4 °C, and the supernatant was collected as the nuclear fraction. Protein concentration was measured using bicinchoninic acid (BCA) protein concentration assay (Pierce) according to the manufacturer’s instructions. Protein samples were made up to a final concentration of 5 μg/μL using relevant buffers and loading buffer (50 mM Tris, 10% glycerol, 2% SDS, 0.1% Bromophenol blue, and 0.2% β-mercaptoethanol). The samples were boiled for 10 min at 95 °C and stored at −20 °C.

### Western blotting

For whole-cell extracts, cells and colonic tissue were lysed using RIPA buffer (50 mM Tris HCl pH7.4, 150 mM NaCl, 0.1% SDS, 1% NP-40, 1% deoxycholate, 1 μM DTT, and protease inhibitor cocktail (Roche)). Following electrophoresis, samples were transferred onto PVDF membranes and blocked in 5% BSA dissolved in PBS and 0.1% Tween (PBST). Membranes were incubated with primary antibodies (anti-p53 (DO-1, Santa Cruz Biotechnology, SC-126), anti-EGFR (Cell Signaling, #4267), anti-pEGFR (Y1068) (Cell Signaling, #2234), anti-pEGFR (Y1101) (eBioscience, #EM1991), anti-AKT (Cell Signaling, #9272), anti-EGFP (Chromotek, #3H9-20), anti-mCherry (Invitrogen, #M11217), anti-pmTOR (Cell Signaling, #2971), anti-pAKT (Cell Signaling, #4060), anti-pS6K (Cell Signaling, #9204), anti-pERK (Cell Signaling, #4370), anti-CycD1 (Abcam, ab226977), anti-cMyc (Cell Signaling, #5605), anti-E2F1 (Cell Signaling, #3742), anti-SGLT1 (Santa Cruz Biotechnology, SC-20582), anti-SHP1 (Cell Signaling, #3759), anti-DDX31 (Cell Signaling, #8761), anti-actin (Abcam, ab8227), anti-tubulin (Abcam, ab7291), and anti-histone H3 (Santa Cruz Biotechnology, SC-517576)) overnight at 4 °C (all at 1:1000) and HRP-linked secondary antibodies (goat anti-mouse, goat anti-rabbit, chicken anti-goat, goat anti-rat) (all at 1:3000) (Dako) at room temperature for 1 h. Membranes were developed by chemiluminescence using ECL reagent and with ChemiDoc (Bio-Rad). Densitometric measurements of proteins of interest were done using Image J and normalized to loading control. In western blot images where the term ‘loading control’ is used, Bio-Rad stain-free imaging was used and the darkest central band was quantified. Histograms were plotted using GraphPad Prism. Statistical analyses were performed with two-tailed unpaired Student’s t-test for comparisons involving two groups of at least *n* = 3 independent biological replicates. Full, uncropped blots are provided in the Source Data file.

### RNA extraction and cDNA

Total RNA was extracted using the PureLink RNA kit (Invitrogen) as per manufacturer’s instructions and cDNA synthesized using the iScript RT Supermix (Bio-Rad) as per manufacturer’s instructions.

### Quantitative PCR analysis

Real-time quantitative PCR was performed in triplicates with SsoAdvanced Universal SYBR Green Supermix (Bio-Rad) on the CFX384 Real-time system (Bio-Rad). Cycling conditions used were as follows: 95 °C for 15 s, 55 °C for 30 s, and 72 °C for 30 s for 40 cycles. Various reactions were verified by analysis of melt curves. Gene expression was normalized to the housekeeping gene *GAPDH*. Analysis was done using the CFX Maestro Software (Bio-Rad).

### Immunoprecipitation

Whole cell, cytoplasmic and nuclear lysates were incubated with 5 μg of anti-p53 (DO1, Santa Cruz Biotechnology, SC-126), anti-EGFR (Cell Signaling, #4267), or anti-AKT (Cell Signaling, #9272) pre-conjugated to Protein G Dynabeads (Invitrogen) overnight at 4 °C. Supernatant was kept as the unbound fraction. Beads were washed 5 times in wash buffer (10 mM Tris HCl pH7.5, 150 mM NaCl, 0.05% NP-40, 0.5 mM EDTA). To elute immunocomplexes, beads were incubated with 100 μl of 2x SDS-sample buffer (Laemmli) (Invitrogen) and boiled for 15 min at 95 °C. Supernatant was analyzed with western blotting.

### Mass spectrometry and analysis

Whole cell lysates were prepared from HCT116 cells stably expressing select TAD and DBD mutants as described above. The immunoprecipitated protein complexes were eluted with 100 mM glycine (pH 2.8), denatured with 15 % 2,2,2-trifluoroethanol (TFE) (Merck) and resuspended in 100 mM triethylammonium bicarbonate (TEAB) (Sigma), to 100 µL. The samples were reduced in 20 mM tris (2-carboxyethyl) phosphine (TCEP) (Gold Biotechnology) and alkylated in 55 mM 2-chloroacetamide (CAA) (Sigma). Samples were diluted to 250 µL with 100 mM TEAB, and digested with Lys-C (Fujifilm WAKO), followed by trypsin (Promega), at 5 µg each at 37 °C. Digested peptides were acidified to 1% trifluoroacetic acid (TFA) (Sigma), desalted with Oasis HLB 1 cc 10 mg columns (Waters), and dried. Desalted peptides were resuspended in 10 µL of 100 mM TEAB, and labeled with 5 µL of Tandem Mass Tags™ 10-plex (TMT10plex™) (Thermo Scientific) with a different isobaric label per sample. The samples were quenched with 10 mM ammonium formate, were combined, and fractioned with high pH (HpH) C18 10 µm resin (Dr Maisch) in 10 mM ammonium formate with increasing concentration of acetonitrile (ACN, 14%, 18%, 22%, 24%, 27%, 32%, 60%). Fractionated peptides were dried and washed twice with 60% acetonitrile in 0.1% formic acid and vacuum dried. The fractions were resuspended in 2% (v/v) acetonitrile (Merck), 0.5% (v/v) acetic acid (Merck), and 0.06% (v/v) TFA in water and subjected to MS analysis. All steps were performed at room temperature unless stated otherwise.

Analysis was performed using Easy nLC1000 (Thermo) chromatography system coupled with Orbitrap Fusion (Thermo). Each sample was separated in 70 min gradient (0.1% formic acid in water and 99.9% acetonitrile with 0.1% formic acid) using 50 cm × 75 µm ID Easy-Spray column (C18, 2 µm particles, Thermo). Gradient parameters: 2–25% over 50 min, ramped to 60% over 10 min, then to 90% over 2 min and held for 5 min. The following acquisition parameters was applied: data dependent acquisition in positive mode with survey scan on 60,000, scan range of 350–1550 m/z, and automatic gain control (AGC) target of 4e5; maximum injection time (IT) 100 ms; HCD fragmentation at 42% collision energy, MS/MS 50,000 resolution and AGC target of 8e4; isolation window 1 m/z, maximum IT 105 ms.

Peak lists were generated with Proteome Discoverer 2.4 (Thermo Scientific) using Mascot 2.6.1 (Matrix Science) and concatenated forward/decoy Human Uniprot database. Search parameters: MS precursor mass tolerance 30 ppm, MS/MS fragment mass tolerance 0.06 Da, 3 missed cleavages; static modifications: Carboamidomethyl (C); variable modifications: Oxidation (M), Deamidated (NQ), Acetyl N-terminal protein, TMT11plex(N-term), TMT11plex(K). False discovery rate estimation with 2 levels: Strict = FDR 1%, Medium = FDR 5%. Differential analysis was performed in R using the *limma* package 3.6 and hits were defined by p-adjusted value <0.05. The TAD- and DBD-specific data were combined for the analysis and interactors common across all the different TAD and DBD mutants were identified.

### Cycloheximide chase assay

Cells were treated with 100 µg/ml of cycloheximide (Sigma). DMSO was used as a control.

### Plasmid and siRNA transfection

Lipofectamine 3000 (Invitrogen) was used for all plasmid transfections as per manufacturer’s recommendations. Transfection reaction mixtures were incubated at room temperature for 15 min before being added dropwise to cells. Media was changed 5 h post-transfection and samples harvested 24 h later. DharmaFECT (Horizon) was used for siRNA transfections as per manufacturer’s recommendations. Transfection reaction mixtures were incubated at on ice for 10 min before being added dropwise to cells. Media was changed 24 h post-transfection.

### Lentiviral shRNA cloning

Desalted oligonucleotides (IDT) were cloned into pLKO.1 (Addgene #8453) with the Age I/EcoRI sites at the 3’ end of the human U6 promoter. The sequences of the oligonucleotides are as follows:

EGFR B sh (Harvard RNAi consortium,^65^)

Fwd: 5′CCGGAGAATGTGGAATACCTAAGGCTCGAGCCTTAGGTATTCCACATTCTCTTTTTG3′

Rev: 5′AATTCAAAAAAGAATGTGGAATACCTAAGGCTCGAGCCTTAGGTATTCCACATTCT3′

mTOR sh (TRCN0000332888,^66^)

Fwd: 5′CCGGGCTGTGCTACACTACAAACATCTCGAGATGTTTGTAGTGTAGCACAGCTTTTTG3′

Rev: 5′AATTCAAAAAGCTGTGCTACACTACAAACATCTCGAGATGTTTGTAGTGTAGCACAGC3′

Plasmids were propagated in and purified from Stbl2 bacterial cells (Invitrogen) and 5 µg of each plasmid was co-transfected together with the Delta 8.2 (1 µg, Addgene #8455) and CMV-VSVG (1 µg, Addgene #8454) plasmids into HEK-293T using Lipofectamine 3000 (Invitrogen) as described according to manufacturer’s instructions. Virus-containing supernatants were collected at 24, 48, and 72 h after transfection and used for transduction of SAECK and HCT116 cells. Upon transduction, cells were cultured under puromycin selection.

### CETSA

Cells were pelleted and washed twice in cold PBS before being resuspended in 120 μl of PBS and heated at 37 °C, 42 °C, 47 °C, 52 °C, 57 °C, and 64 °C for 3 min in a thermocycler followed by 5 min of cooling on ice. Cells were lysed to obtain cytoplasmic and nuclear fractions as described above.

### Small molecule drug screen

HCT116, MCF7 or H1299 cells were counted and 1000 cells were plated in 50 μl of culture media in 384-well white flat-bottom plates (Corning) and incubated at 37 °C in a humidified atmosphere of 5% CO2 overnight. Each cell line had 3 replicates. The next day, 1 μM of a customized small molecule compound library targeting metabolic pathways (303 compounds, MCE) were added to cells with the Bravo Automated Liquid Handling Platform (Agilent). Cells were then incubated for 72 h at 37 °C in a humidified atmosphere of 5% CO2 before 10 μl of CellTiter-Glo (Promega) reagent was added to each well with the MultiFlo Microplate Dispenser (BioTek). Cells were incubated at room temperature for a minimum of 10 min after which luminescence readings were recorded by an Infinite M1000 Microplate Reader (Tecan) or GloMax Plate Reader (Promega).

The survival rate per drug for each sample was determined by taking the means of the sample replicates. Drugs that gave <50% survival rate in at least one sample were retained. The resulting data was then used to generate a heatmap using the R package gplots^67^.

### Drug dose response analyses

The following drugs were used: Erlotinib (MCE, HY-50896), Torin 1 (MCE, HY-13003), GSK 690693 (MCE, HY-10249), and PI 103 (MCE, HY-10115). HCT116, MCF7, or H1299 cells were counted and 1000 cells (in triplicates) were plated in 50 μl of culture media in 384-well white flat-bottom plates (Corning) and incubated at 37 °C in a humidified atmosphere of 5% CO2 overnight. Drugs were diluted to a final concentration of 0.1 nM to 20 μM. Cells were then incubated for 72 h at 37 °C in a humidified atmosphere of 5% CO2 before 10 μl of CellTiter-Glo reagent was added to each well with the MultiFlo Microplate Dispenser (BioTek). Cells were incubated at room temperature for a minimum of 10 min after which luminescence readings were recorded by an Infinite M1000 Microplate Reader (Tecan) or GloMax Plate Reader (Promega). Raw luminescence readings were normalized to DMSO luminescence readings and plotted on GraphPad Prism. Curve fitting was done with [Inhibitor] vs. response–Variable slope (four parameters) and Bottom = 0 Top = 100 constraints applied. Statistical analyses were performed with two-tailed unpaired Student’s *t* test for comparisons involving two groups.

### Colony formation assay

Cells were plated at 300 cells/well (HCT116, H1299, and MCF7) and 700 cells/well (SAECK) in 6-well plates in triplicate. After 12–14 days, colonies were fixed with ice cold methanol for 10 minutes on ice and stained with 0.1% crystal violet solution for 2 h at room temperature. For quantitative analysis, colonies were de-stained by incubation with 10% acetic acid for 15 minutes at room temperature (under agitation) and absorbance at 590 nm was measured.

### Protein modeling

Models of the putative complexes formed between p53TAD and EGFR and between p53DBD and EGFR were generated using either the experimental structure (EGFR kinase domain, p53TAD) or snapshots from computer simulations of the p53DBD. The kinase domain of EGFR (residues from 669 to 960, PDBID: 1M14), two alpha helical regions of the p53 TAD (residues from 13 to 27, 46 to 56, PDBID: 2L14) and the whole DBD (residues from 94 to 291, PDBID: 2AHI) were used for in-silico docking calculations; models of the DBD mutant (R175H, I255S and R248W) structures were generated by carrying out Molecular Dynamics simulations. Mutations were introduced in the p53DBD wildtype (WT) structure (PDB: 2AHI) in Pymol and were subjected to MD simulations. The Xleap module of AMBER 18 was used to prepare the systems for the MD simulations. Each simulation system was neutralized with appropriate numbers of counter ions and each neutralized system was solvated in an octahedral box with TIP3P water molecules, leaving at least 10 Å between the solute atoms and the boundaries of the box. MD simulations were carried out with the pmemd.cuda module of the AMBER 18 package in combination with the ff14SB force field. MD simulations were carried out in explicit solvent at 300 K. During the simulations the long-range electrostatic interactions were treated with the particle mesh Ewald method using a real space cut off distance of 9 Å. The settle algorithm was used to constrain bond vibrations involving hydrogen atoms, which allowed a time step of 2 fs during the simulations. Solvent molecules and counter ions were initially relaxed using energy minimization with restraints on the protein atoms. This was followed by unrestrained energy minimization to remove any steric clashes. Subsequently the system was gradually heated from 0 to 300 K using MD simulations with positional restraints (force constant: 50 kcal mol-1 Å−2) on the non-hydrogen protein atoms over a period of 0.25 ns allowing water molecules and ions to move freely followed by gradual removal of the positional restraints and a 2 ns unrestrained equilibration of the whole system at 300 K. The resulting system was used as the starting structure for the production phase and three independent (using different initial random velocities) MD simulations were carried out for 100 ns each. Accelerated MD simulations (aMD) with dual boost potential were used to further enhance the conformational sampling of the p53 DBDs. Simulation trajectories were visualized using VMD and figures were generated using Pymol. To generate the models of the complexes, we used the program HADDOCK with standard parameters^68^. The program requires as its input the structures of the two molecules whose complexes are desired and if possible, the definitions of the regions that are thought to be the interfaces.

### Reactome

Differentially regulated genes in TAD vs DBD and DBD vs TAD were analyzed using Reactome (https://reactome.org/).

### Reporting summary

Further information on research design is available in the Nature Portfolio Reporting Summary linked to this article.

## Supplementary information

Supplementary Information

Description of Additional Supplementary Files

Supplementary Data 1

Reporting Summary

## Data Availability

The raw mass spectra and search data have been deposited to the ProteomeXchange Consortium via the jPOST partner repository with the data set identifier PXD031725 [http://proteomecentral.proteomexchange.org/cgi/GetDataset?ID=PXD031725]. Source data are provided with this paper.

## Source data

Source Data
