# Supplementary material for: Domain-specific p53 mutants activate EGFR by distinct mechanisms exposing tissue-independent therapeutic vulnerabilities
Source: Nat Commun. 2023 Mar 28;14:1726. doi: 10.1038/s41467-023-37223-3 (PMC10050071; doi:10.1038/s41467-023-37223-3)
Supplement: Supplementary file 1 — Supplementary Information [file 41467_2023_37223_MOESM1_ESM.pdf]

Supplementary Information

Domain-specific p53 mutants activate EGFR by distinct mechanisms exposing tissue-independent therapeutic vulnerabilities

Teresa Lai Fong Ho[1,2], May Yin Lee[3], Hui Chin Goh[2], Germaine Yi Ning Ng[4], Jane Jia Hui Lee[3], Srinivasaraghavan Kannan[5], Yan Ting Lim[6,7], Tianyun Zhao[6,7], Edwin Kok Hao Lim[3], Cheryl Zi Jin Phua[3], Yi Fei Lee[3], Rebecca Yi Xuan Lim[3], Perry Jun Hao Ng[3], Ju Yuan[3], Dedrick Kok Hong Chan[8,9], Bettina Lieske[9,10], Choon Seng Chong[9,10], Kuok Chung Lee[9], Jeffrey Lum[11], Wai Kit Cheong[9], Khay Guan Yeoh[12], Ker Kan Tan[9,10], Radoslaw M. Sobota[6,7], Chandra S. Verma[5,13,14], David P. Lane[1], Wai Leong Tam[2,3,13,15,16] and Ashok R. Venkitaraman[1,2,16,*].

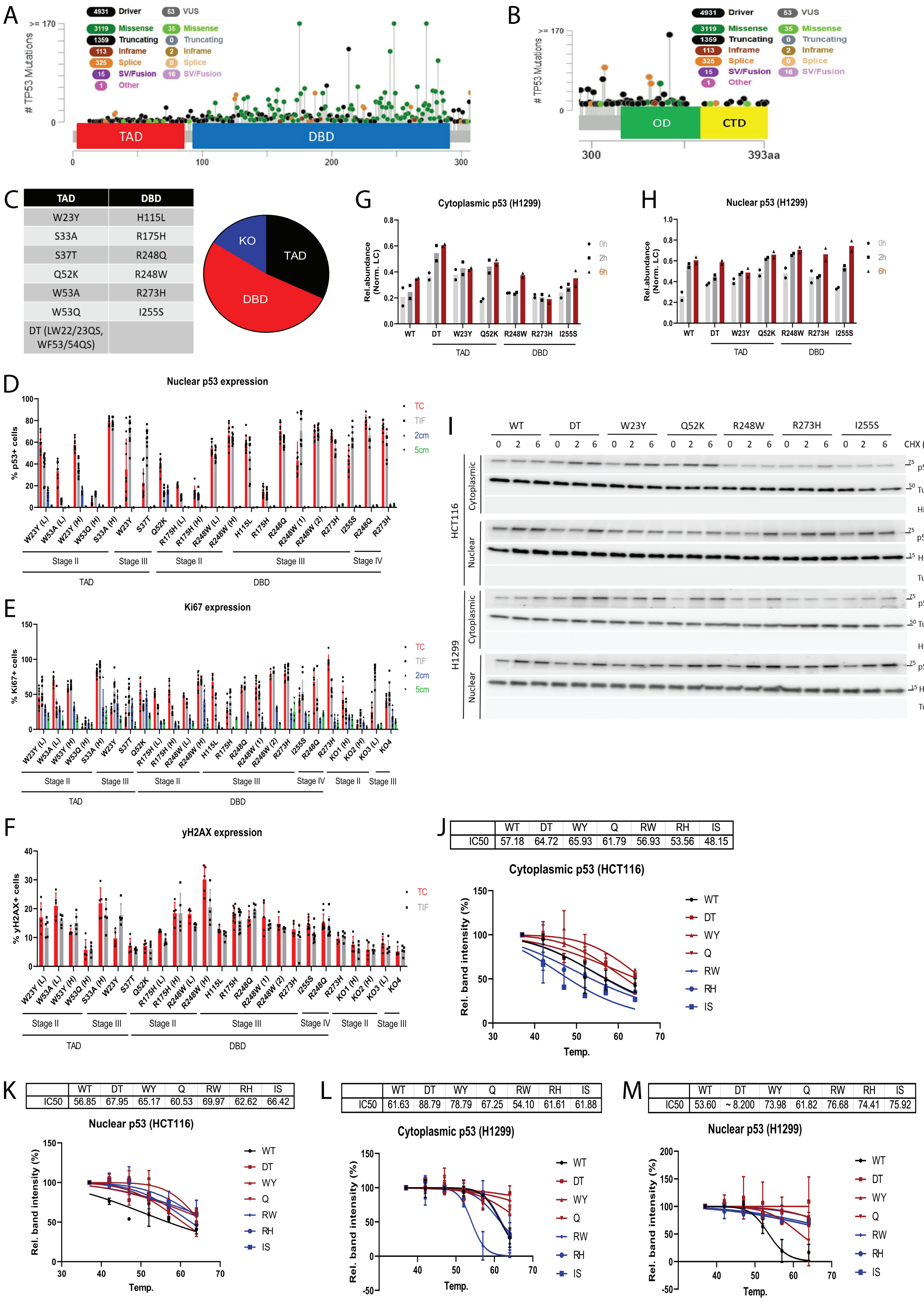
A
TP53 Mutations
4931 Driver
53 VUS
3119 Missense
35 Missense
1359 Truncating
0 Truncating
113 Inframe
2 Inframe
325 Splice
0 Splice
15 SV/Fusion
16 SV/Fusion
1 Other
TAD
DBD
B
OD
CTD
300
393aa
C
TAD | DBD
W23Y | H115L
S33A | R175H
S37T | R248Q
Q52K | R248W
W53A | R273H
W53Q | I255S
DT (LW22/23QS, WF53/54QS)
KO
TAD
DBD
G
Cytoplasmic p53 (H1299)
Rel.abundance (Norm. LC)
H
Nuclear p53 (H1299)
0h
2h
6h
D
Nuclear p53 expression
% p53+ cells
TC
TIF
2cm
5cm
Stage II
Stage III
Stage IV
E
Ki67 expression
% Ki67+ cells
F
yH2AX expression
% yH2AX+ cells
I
WT DT W23Y Q52K R248W R273H I255S
CHX (h)
HCT116
H1299
Cytoplasmic
Nuclear
p53-EGFP
Tubulin
HistH3
J
IC50 | WT 57.18 | DT 64.72 | WY 65.93 | Q 61.79 | RW 56.93 | RH 53.56 | IS 48.15
Cytoplasmic p53 (HCT116)
K
IC50 | WT 56.85 | DT 67.95 | WY 65.17 | Q 60.53 | RW 69.97 | RH 62.62 | IS 66.42
Nuclear p53 (HCT116)
L
IC50 | WT 61.63 | DT 88.79 | WY 78.79 | Q 67.25 | RW 54.10 | RH 61.61 | IS 61.88
Cytoplasmic p53 (H1299)
M
IC50 | WT 53.60 | DT ~ 8.200 | WY 73.98 | Q 61.82 | RW 76.68 | RH 74.41 | IS 75.92
Nuclear p53 (H1299)
Rel. band intensity (%)
Temp.

Supplementary Fig. 1: Phenotypic differences between TAD and DBD *TP53* mutant cells in vivo and in vitro

(**A-B**) Frequency of various types of *TP53* mutations as reported in pan-cancer clinical samples. Data from MSK-IMPACT Clinical Sequencing Cohort consisting of 10336 patients and 10945 samples. (**C**) Proportion of colorectal tumors expressing either TAD or DBD mutations or lacking p53 (KO) in our patient cohort. Table lists the unique TAD and DBD mutations identified. (**D-F**) Quantification of the percentage of cells with (D) nuclear p53, (E) Ki67 and (F) γH2AX staining in TAD and DBD mutant colorectal tumors from 10 fields of view (Mean ± SEM). (TC: tumor core; TIF: tumor invasive front) and surrounding normal tissue (2cm and 5cm from the tumor invasive front). (**G-H**) p53 protein turnover in H1299 (G) cytosolic and (H) nuclear cellular fractions following cycloheximide treatment. n=2. (**I**) Protein levels of p53-EGFP (cytosolic and nuclear cellular fractions) in untreated and cycloheximide treated HCT116 and H1299 expressing WT and mutant p53. n=2. (**J-M**) CETSA-derived curves of cytosolic and nuclear WT and mutant p53 in untreated HCT116 and H1299 cells. IC50 values represent temperature at which 50% of the protein has degraded. Cytosolic TAD (n=3 independent mutants) versus DBD (n=3 independent mutants) $p<0.001$. n=2. Two-tailed unpaired Student's t-test was performed. (* $p<0.05$, ** $p<0.01$, *** $p<0.001$, **** $p<0.0001$). Source data are provided as a Source Data file.

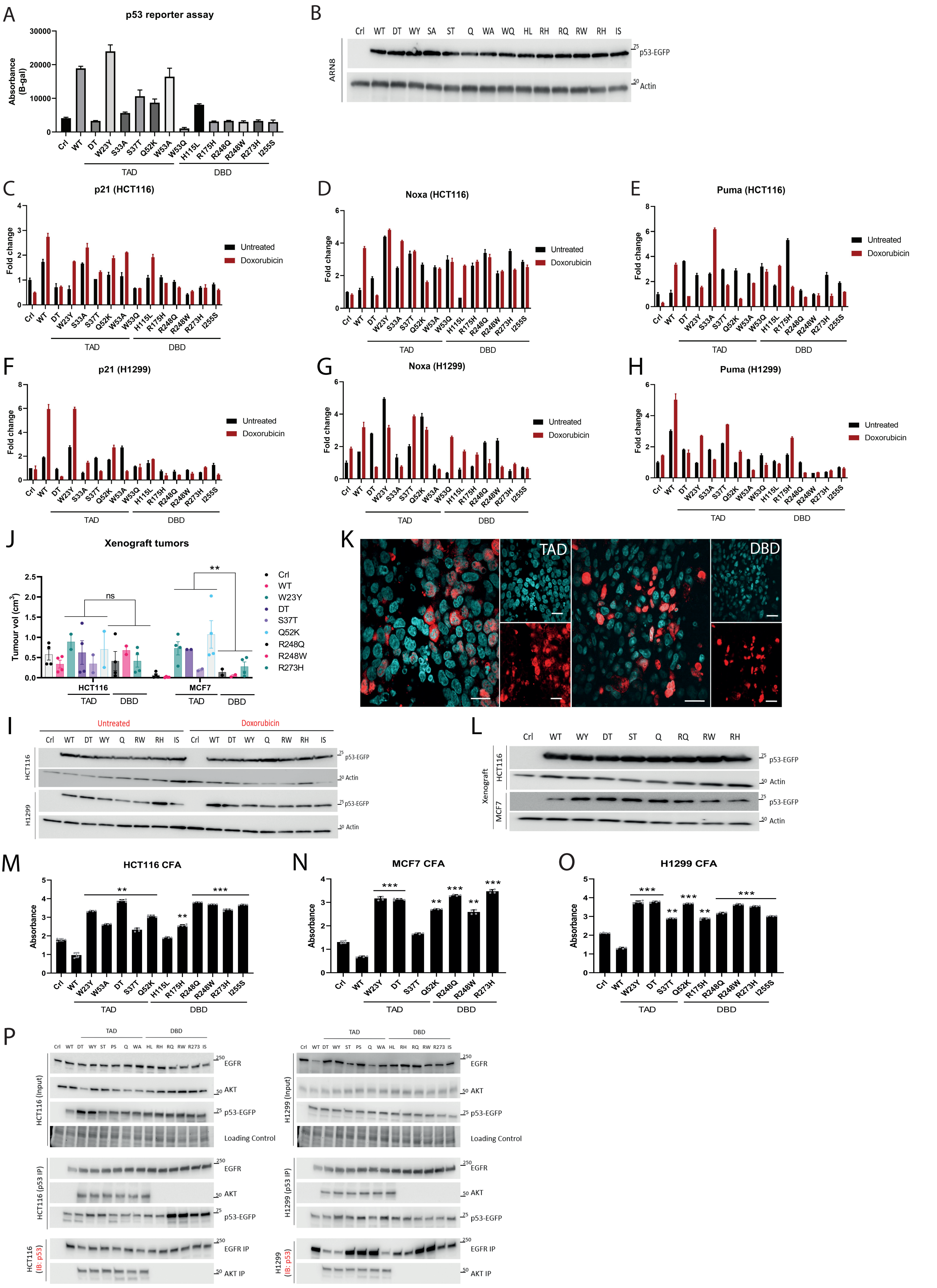

A
p53 reporter assay
Absorbance (B-gal)
Crl WT DT W23Y S33A S37T Q52K W53A W53Q H115L R175H R248Q R248W R273H I255S
TAD
DBD
B
Crl WT DT WY SA ST Q WA WQ HL RH RQ RW RH IS
ARN8
p53-EGFP
Actin
C
p21 (HCT116)
D
Noxa (HCT116)
E
Puma (HCT116)
F
p21 (H1299)
G
Noxa (H1299)
H
Puma (H1299)
Fold change
Untreated
Doxorubicin
J
Xenograft tumors
Tumour vol (cm3)
ns
**
HCT116
MCF7
Crl
WT
W23Y
DT
S37T
Q52K
R248Q
R248W
R273H
K
TAD
DBD
I
Untreated
Doxorubicin
Crl WT DT WY Q RW RH IS
HCT116
H1299
p53-EGFP
Actin
L
Crl WT WY DT ST Q RQ RW RH
Xenograft
HCT116
MCF7
p53-EGFP
Actin
M
HCT116 CFA
N
MCF7 CFA
O
H1299 CFA
Absorbance
P
Crl WT DT WY ST PS Q WA HL RH RQ RW R273 IS
HCT116 (Input)
H1299 (Input)
EGFR
AKT
p53-EGFP
Loading Control
HCT116 (p53 IP)
H1299 (p53 IP)
HCT116 (IB: p53)
H1299 (IB: p53)
EGFR IP
AKT IP

Upregulated in TAD

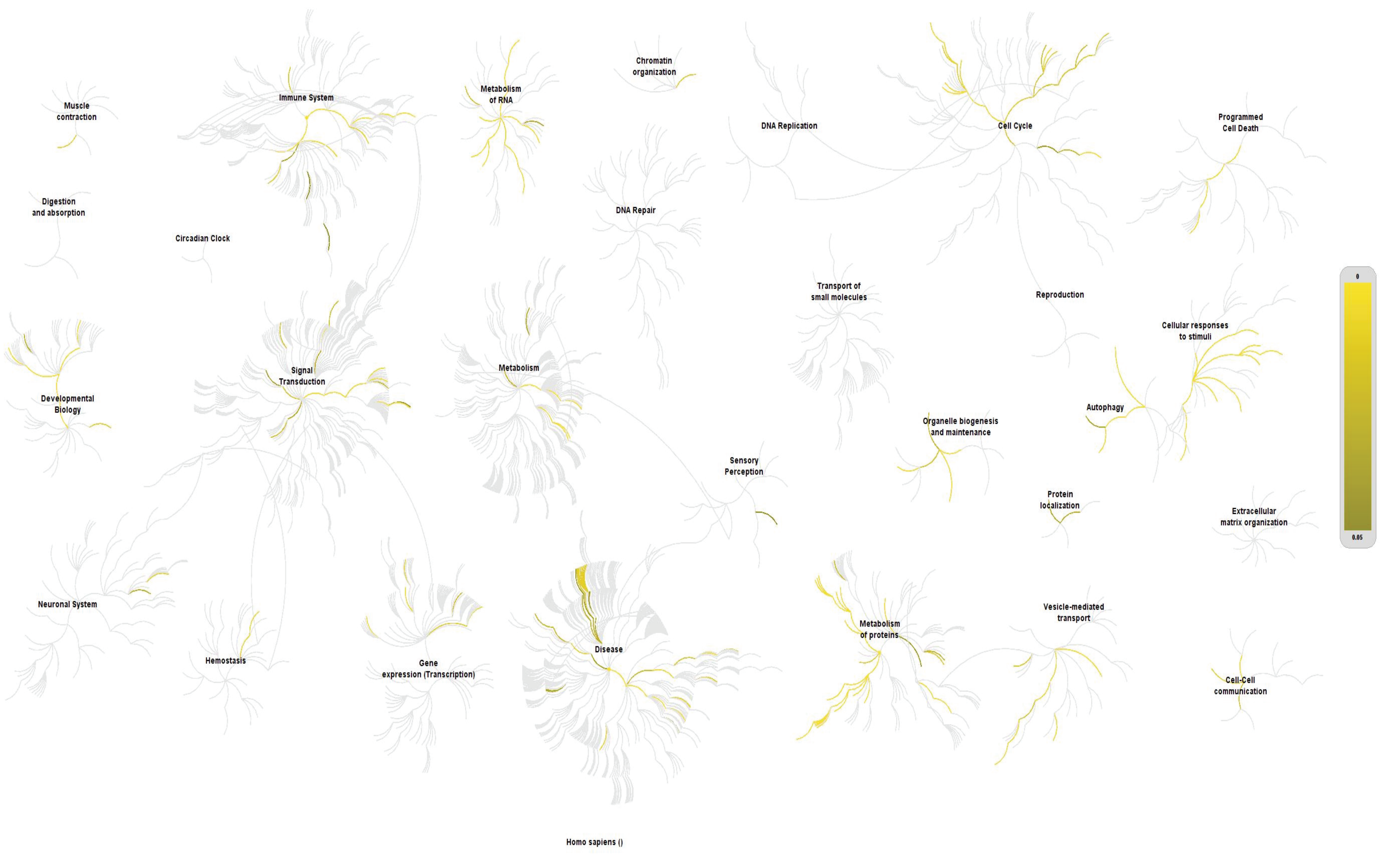

Upregulated in DBD

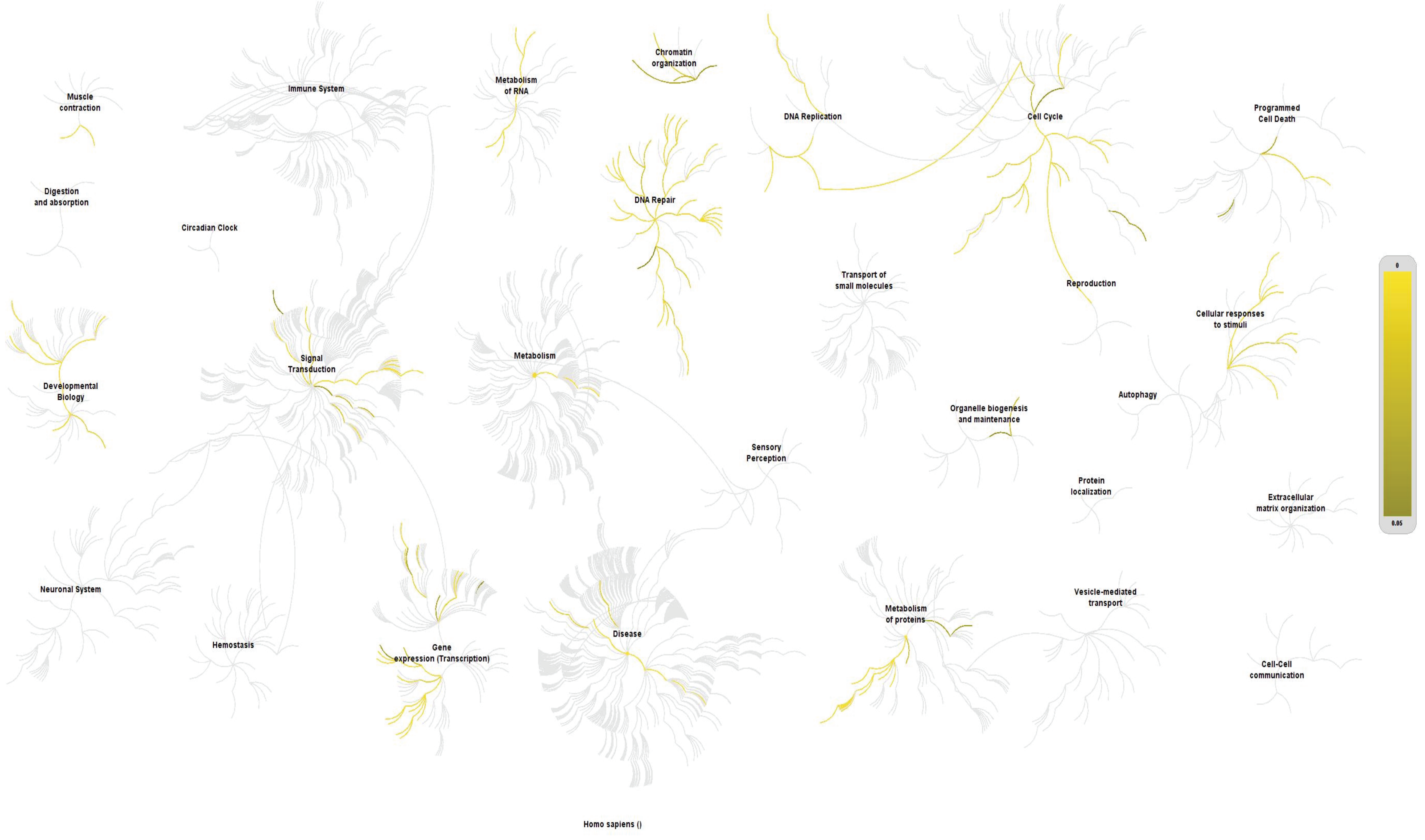

Supplementary Fig. 2: Phenotypic differences between TAD and DBD *TP53* mutant cells in vivo and in vitro

(**A**) Quantification of Beta-Galactosidase activity in p53 promoter assay with ARN8 cells expressing WT and mutant p53. n=12 independent replicates. (**B**) Protein levels of p53-EGFP in ARN8 cells transiently expressing WT and mutant p53. n=2. (**C-H**) Quantification of mRNA transcript levels of (C, F) *p21*, (D, G) *NOXA* and (E, H) *PUMA* in untreated and doxorubicin treated HCT116 and H1299 cells expressing WT and mutant p53. n=12 independent replicates. (**I**) Protein levels of p53-EGFP in untreated and doxorubicin treated HCT116 and H1299 cells expressing WT and mutant p53. n=2. (**J**) Quantification of tumor volume of xenograft tumors derived from HCT116 and MCF7 cells expressing WT and mutant p53. n=2 mice; n=4 tumors per mutant. Individual data points denote individual tumors. Statistical tests performed on TAD versus DBD. (**K**) Representative immunofluorescence staining of p53 (red) in DBD and TAD mutant xenograft tumor tissue. DAPI staining shows nuclei. n=2. Scale bar = 30µm. (**L**) Protein levels of p53-EGFP in lines derived from respective HCT116 and MCF7 xenograft tumors. n=1-2 mice, n=2-4 tumors per mutant. (**M-O**) Quantification of crystal violet intensity from colony formation assays using HCT116, MCF7 and H1299 cells constitutively expressing WT and mutant p53. Statistical tests performed on TAD/DBD (n=3 independent mutants each) versus Control. n=4 independent experiments. (**P**) Protein levels of EGFR, AKT and p53-EGFP in input fractions from HCT116 and H1299 cells expressing WT and mutant p53. P53 IP and reverse IP validation of EGFR and AKT. n=2. (**Q-R**) Reactome plots of protein interactors enriched in (Q) TAD- and (R) DBD- mutant expressing HCT116 cells respectively indicating the molecular and functional pathways that they are involved in. Two-tailed unpaired Student's t-test was performed. (* $p<0.05$, ** $p<0.01$, *** $p<0.001$, **** $p<0.0001$). Source data are provided as a Source Data file.

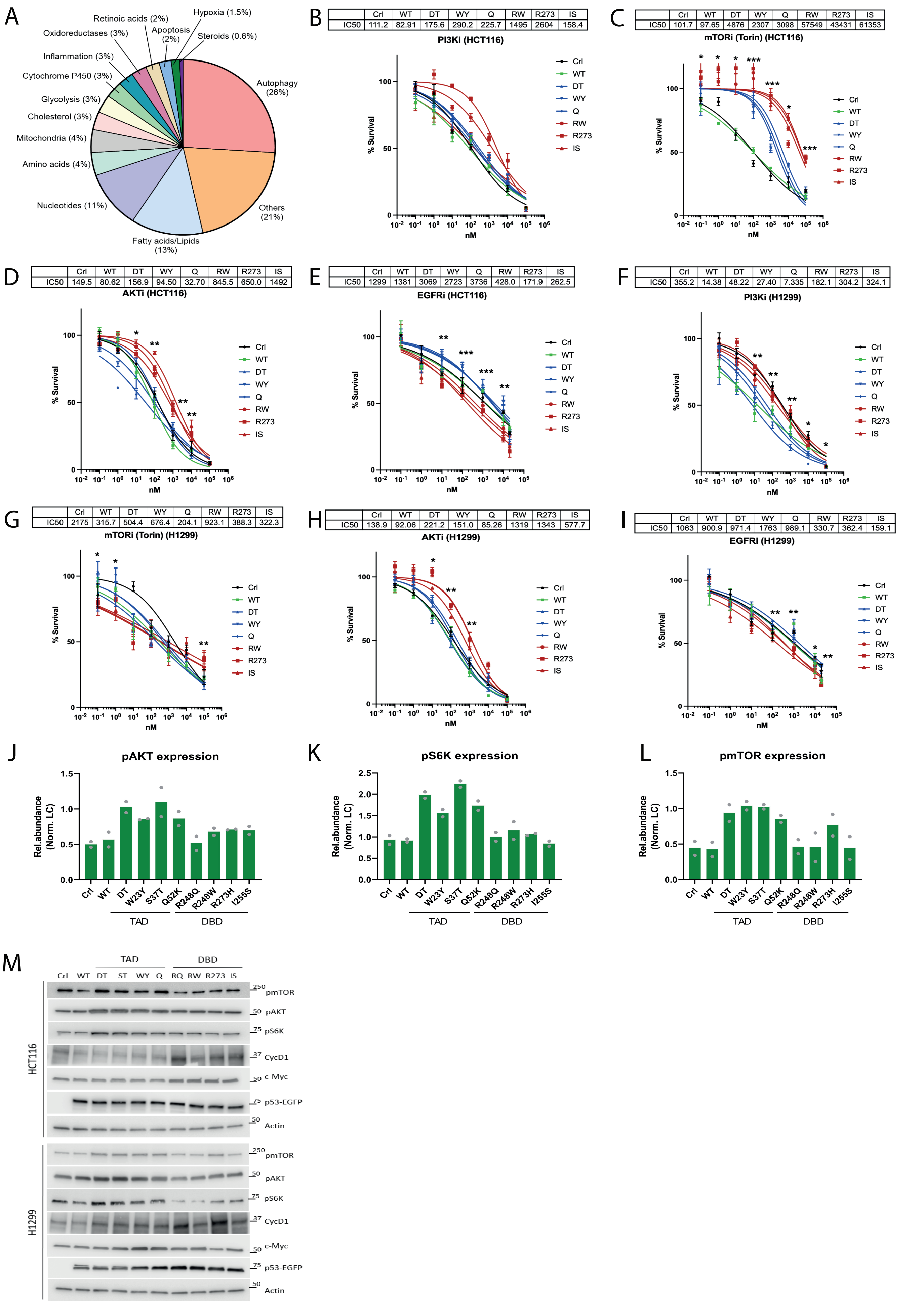
A
Retinoic acids (2%)
Hypoxia (1.5%)
Oxidoreductases (3%)
Apoptosis (2%)
Steroids (0.6%)
Inflammation (3%)
Cytochrome P450 (3%)
Glycolysis (3%)
Cholesterol (3%)
Mitochondria (4%)
Amino acids (4%)
Nucleotides (11%)
Fatty acids/Lipids (13%)
Others (21%)
Autophagy (26%)
B
| Crl | WT | DT | WY | Q | RW | R273 | IS
IC50 | 111.2 | 82.91 | 175.6 | 290.2 | 225.7 | 1495 | 2604 | 158.4
PI3Ki (HCT116)
C
| Crl | WT | DT | WY | Q | RW | R273 | IS
IC50 | 101.7 | 97.65 | 4876 | 2307 | 3098 | 57549 | 43431 | 61353
mTORi (Torin) (HCT116)
D
| Crl | WT | DT | WY | Q | RW | R273 | IS
IC50 | 149.5 | 80.62 | 156.9 | 94.50 | 32.70 | 845.5 | 650.0 | 1492
AKTi (HCT116)
E
| Crl | WT | DT | WY | Q | RW | R273 | IS
IC50 | 1299 | 1381 | 3069 | 2723 | 3736 | 428.0 | 171.9 | 262.5
EGFRi (HCT116)
F
| Crl | WT | DT | WY | Q | RW | R273 | IS
IC50 | 355.2 | 14.38 | 48.22 | 27.40 | 7.335 | 182.1 | 304.2 | 324.1
PI3Ki (H1299)
G
| Crl | WT | DT | WY | Q | RW | R273 | IS
IC50 | 2175 | 315.7 | 504.4 | 676.4 | 204.1 | 923.1 | 388.3 | 322.3
mTORi (Torin) (H1299)
H
| Crl | WT | DT | WY | Q | RW | R273 | IS
IC50 | 138.9 | 92.06 | 221.2 | 151.0 | 85.26 | 1319 | 1343 | 577.7
AKTi (H1299)
I
| Crl | WT | DT | WY | Q | RW | R273 | IS
IC50 | 1063 | 900.9 | 971.4 | 1763 | 989.1 | 330.7 | 362.4 | 159.1
EGFRi (H1299)
% Survival
nM
Crl
WT
DT
WY
Q
RW
R273
IS
J
pAKT expression
K
pS6K expression
L
pmTOR expression
Rel.abundance (Norm. LC)
Crl WT DT W23Y S37T Q52K R248Q R248W R273H I255S
TAD
DBD
M
TAD
DBD
Crl WT DT ST WY Q RQ RW R273 IS
HCT116
H1299
pmTOR
pAKT
pS6K
CycD1
c-Myc
p53-EGFP
Actin

Supplementary Fig. 3: Differential response to small molecule inhibitors

(**A**) Summary of other signaling and metabolic pathways and/or molecular functions that TAD and DBD mutant cells are differentially dependent on based on sensitivity to small molecular inhibitor treatment. (**B-I**) Dose response curves of HCT116 and H1299 cells expressing WT and mutant p53 treated with inhibitors targeting (B, F) PI3K, (C, G) mTOR, (D, H) AKT and (E, I) EGFR. n=3 independent experiments. (**J-L**) Quantification of protein levels of (J) pAKT, (K) pS6K and (L) pmTOR in H1299 cells expressing WT and mutant p53. n=2. (**M**) Protein levels of pmTOR, pAKT, pS6K, pERK, Cyclin D1 and c-Myc in HCT116 and H1299 expressing WT and mutant p53. n=2. Two-tailed unpaired Student's t-test was performed. (* $p<0.05$, ** $p<0.01$, *** $p<0.001$, **** $p<0.0001$). Source data are provided as a Source Data file.

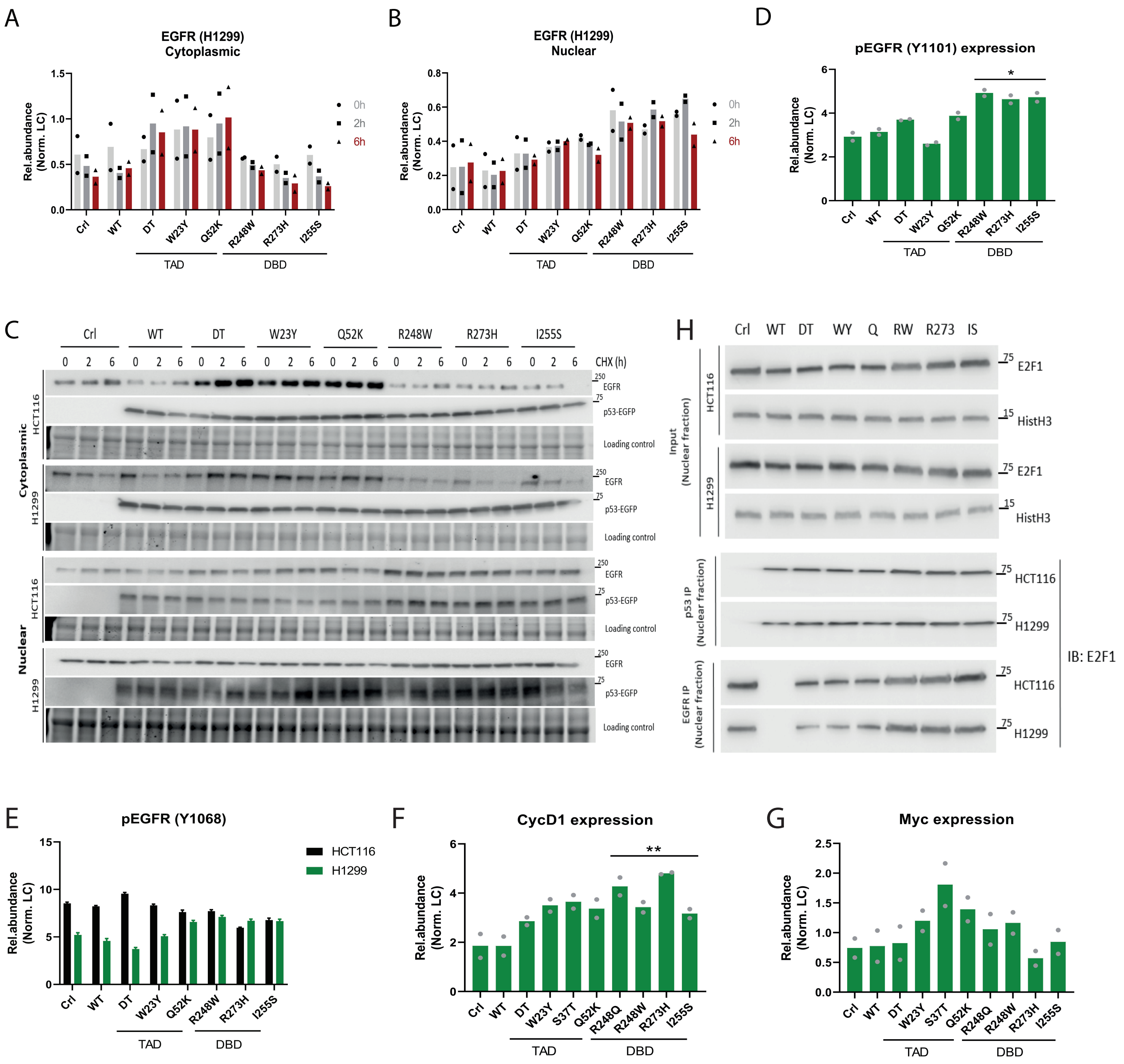
A
EGFR (H1299)
Cytoplasmic
Rel.abundance (Norm. LC)
0h
2h
6h
Crl
WT
DT
W23Y
Q52K
R248W
R273H
I255S
TAD
DBD
B
EGFR (H1299)
Nuclear
Rel.abundance (Norm. LC)
0h
2h
6h
D
pEGFR (Y1101) expression
Rel.abundance (Norm. LC)
*
C
CHX (h)
Cytoplasmic
Nuclear
HCT116
H1299
EGFR
p53-EGFP
Loading control
H
Crl WT DT WY Q RW R273 IS
Input (Nuclear fraction)
p53 IP (Nuclear fraction)
EGFR IP (Nuclear fraction)
E2F1
HistH3
IB: E2F1
E
pEGFR (Y1068)
HCT116
H1299
Rel.abundance (Norm. LC)
F
CycD1 expression
Rel.abundance (Norm. LC)
**
S37T
R248Q
G
Myc expression
Rel.abundance (Norm. LC)

Supplementary Fig. 4: Comparison of EGFR stability and signaling in TAD and DBD mutant cells

(**A-B**) Quantification of EGFR protein turnover in H1299 (A) cytosolic and (B) nuclear cellular fractions following treatment with cycloheximide. n=2. (**C**) Protein levels of cytosolic and nuclear EGFR in HCT116 and H1299 cells expressing WT and mutant p53 treated with cycloheximide. n=2. (**D**) Quantification of pEGFR (Y1101) protein levels in H1299 cells expressing WT and mutant p53. Statistical tests performed on TAD (n=3 independent mutants) versus DBD (n=3 independent mutants). n=2. (**E**) Quantification of pEGFR (Y1068) protein levels in HCT116 and H1299 cells expressing WT and mutant p53. n=2. (**F-G**) Quantification of (F) Cyclin D1 and (G) c-Myc protein levels in H1299 cells expressing WT and mutant p53. Statistical tests performed on TAD (n=3 independent mutants) versus DBD (n=3 independent mutants). n=2. (**H**) Protein levels of nuclear E2F1 in input fractions from HCT116 and H1299 cells expressing WT and mutant p53. P53 and EGFR IP of nuclear fractions. n=2. Two-tailed unpaired Student's t-test was performed. (* $p<0.05$, ** $p<0.01$, *** $p<0.001$, **** $p<0.0001$). Source data are provided as a Source Data file.

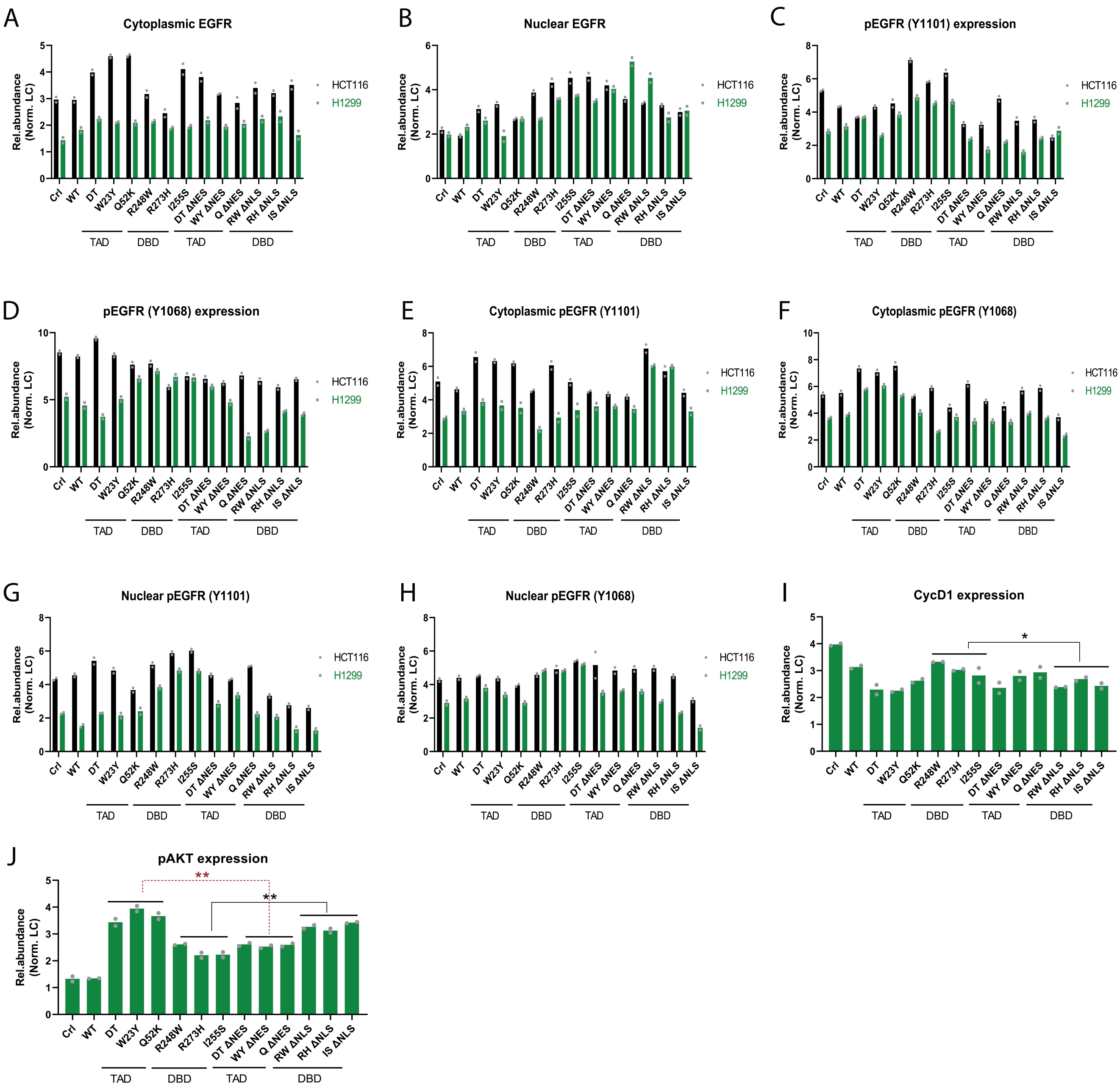
A
Cytoplasmic EGFR
B
Nuclear EGFR
C
pEGFR (Y1101) expression
D
pEGFR (Y1068) expression
E
Cytoplasmic pEGFR (Y1101)
F
Cytoplasmic pEGFR (Y1068)
G
Nuclear pEGFR (Y1101)
H
Nuclear pEGFR (Y1068)
I
CycD1 expression
J
pAKT expression
Rel.abundance (Norm. LC)
HCT116
H1299
Crl
WT
DT
W23Y
Q52K
R248W
R273H
I255S
DT ΔNES
WY ΔNES
Q ΔNES
RW ΔNLS
RH ΔNLS
IS ΔNLS
TAD
DBD
*
**

Supplementary Fig. 5: Differential stabilization of EGFR in the cytosol and nucleus in TAD and DBD mutant cells

(**A-D**) Quantification of (A) cytosolic EGFR, (B) nuclear EGFR, (C) pEGFR (Y1101) and (D) pEGFR (Y1068) protein levels in HCT116 and H1299 cells expressing WT and mutant p53. n=2. (**E-H**) Quantification of (E, F) cytosolic and (G, H) nuclear pEGFR protein levels in HCT116 and H1299 cells expressing WT and mutant p53. n=2. (**I-J**) Quantification of (I) Cyclin D1 and (J) pAKT protein levels in HCT116 cells expressing WT and mutant p53. Statistical tests performed on TAD (n=3 independent mutants) versus DBD (n=3 independent mutants). n=2. Two-tailed unpaired Student's t-test was performed. (* $p<0.05$, ** $p<0.01$, *** $p<0.001$, **** $p<0.0001$). Source data are provided as a Source Data file.

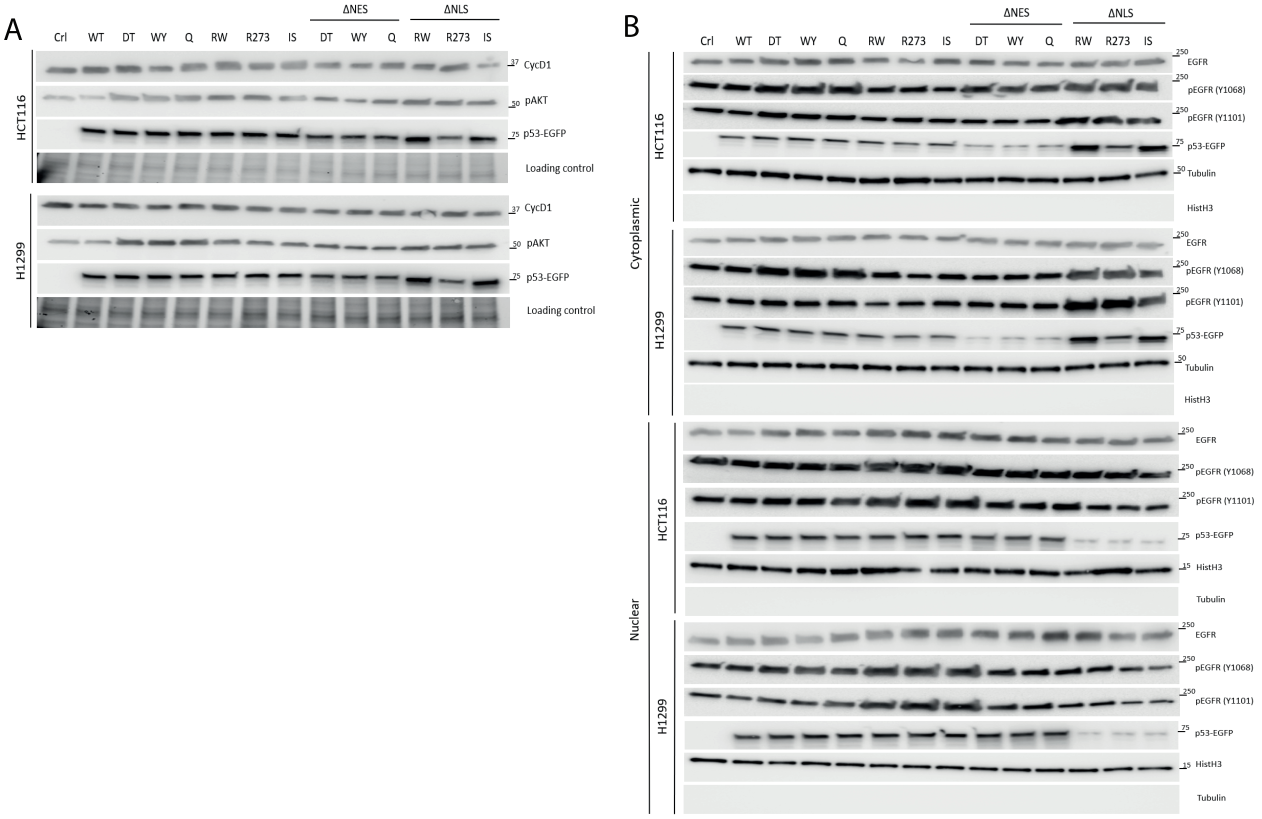

A
ΔNES
ΔNLS
Crl
WT
DT
WY
Q
RW
R273
IS
DT
WY
Q
RW
R273
IS
HCT116
H1299
CycD1
pAKT
p53-EGFP
Loading control
37
50
75
B
Cytoplasmic
Nuclear
EGFR
pEGFR (Y1068)
pEGFR (Y1101)
Tubulin
HistH3
250
15

Supplementary Fig. 6: EGFR activation in DBD nuclear localization and TAD nuclear export defective mutants

(**A**) Protein levels of various factors in HCT116 and H1299 cells expressing WT and mutant p53. n=2. (**B**) Protein levels of various factors in cytosolic and nuclear fractions of HCT116 and H1299 cells expressing WT and mutant p53. n=2. Source data are provided as a Source Data file.

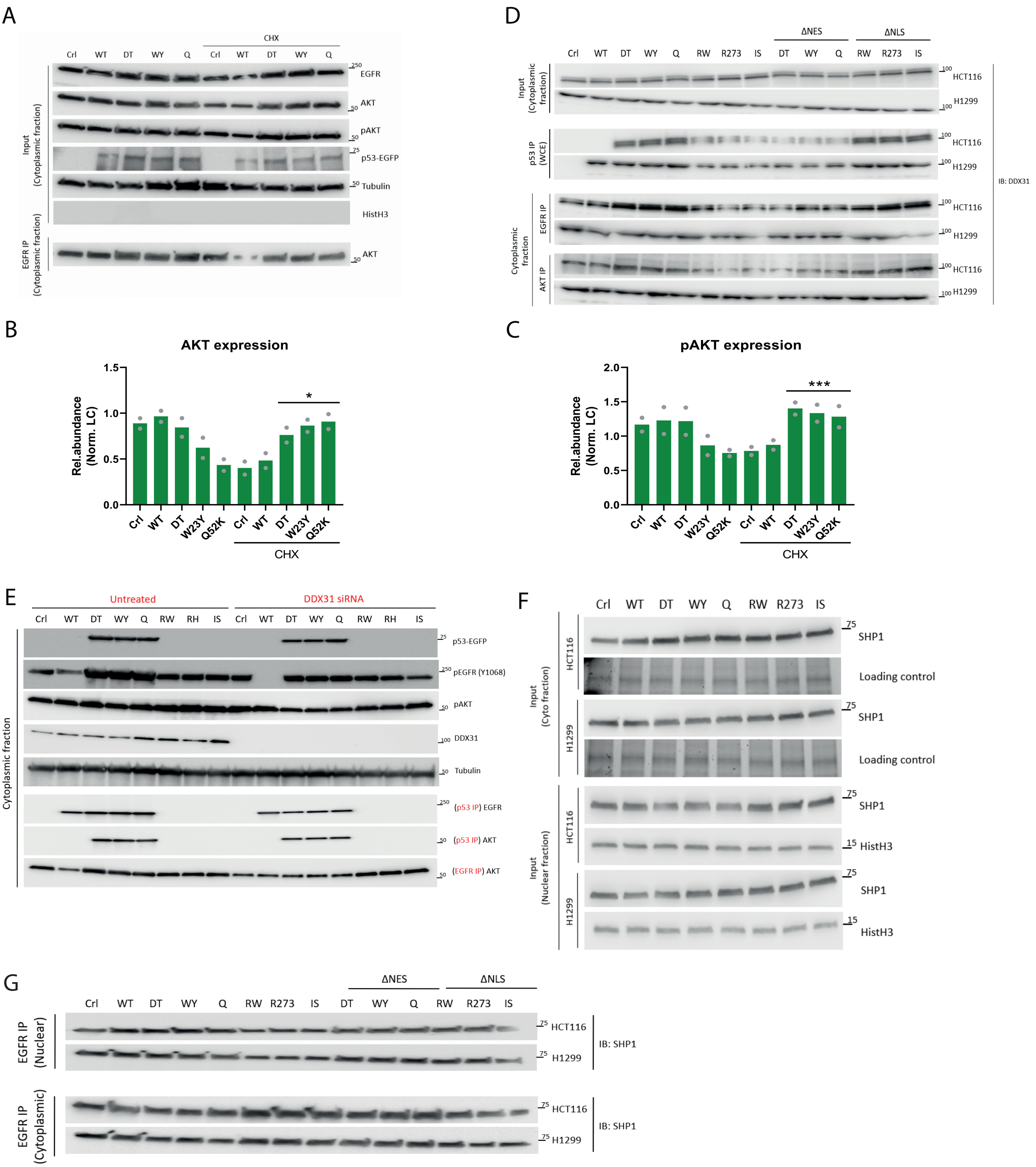
A
CHX
Crl WT DT WY Q Crl WT DT WY Q
EGFR
AKT
pAKT
p53-EGFP
Tubulin
HistH3
AKT
Input (Cytoplasmic fraction)
EGFR IP (Cytoplasmic fraction)
D
ΔNES
ΔNLS
Crl WT DT WY Q RW R273 IS DT WY Q RW R273 IS
HCT116
H1299
Input (Cytoplasmic fraction)
p53 IP (WCE)
Cytoplasmic fraction
EGFR IP
AKT IP
IB: DDX31
B
AKT expression
Rel.abundance (Norm. LC)
1.5
1.0
0.5
0.0
Crl WT DT W23Y Q52K Crl WT DT W23Y Q52K
CHX
*
C
pAKT expression
Rel.abundance (Norm. LC)
2.0
1.5
1.0
0.5
0.0
Crl WT DT W23Y Q52K Crl WT DT W23Y Q52K
CHX
***
E
Untreated
DDX31 siRNA
Crl WT DT WY Q RW RH IS Crl WT DT WY Q RW RH IS
p53-EGFP
pEGFR (Y1068)
pAKT
DDX31
Tubulin
(p53 IP) EGFR
(p53 IP) AKT
(EGFR IP) AKT
Cytoplasmic fraction
F
Crl WT DT WY Q RW R273 IS
SHP1
Loading control
HistH3
HCT116
H1299
Input (Cyto fraction)
Input (Nuclear fraction)
G
ΔNES
ΔNLS
Crl WT DT WY Q RW R273 IS DT WY Q RW R273 IS
HCT116
H1299
EGFR IP (Nuclear)
EGFR IP (Cytoplasmic)
IB: SHP1

Supplementary Fig. 7: Stabilization of AKT in TAD mutants and disruption of SHP1-EGFR interaction in DBD mutants

(**A**) Protein levels of EGFR, AKT and pAKT in input cytosolic fractions from H1299 cells expressing TAD mutants under untreated and cycloheximide-treated conditions. Protein levels of AKT pulled down with EGFR. n=2. (**B-C**) Quantification of (B) AKT and (C) pAKT protein levels in H1299 cells expressing TAD mutants under untreated and cycloheximide-treated conditions. Statistical tests performed on TAD (n=3 independent mutants) versus TAD+CHX (n=3 independent mutants). n=2. (**D**) Protein levels of DDX31 in input cytosolic fractions from HCT116 and H1299 cells expressing WT and mutant p53. Protein levels of DDX31 pulled down with p53, EGFR and AKT respectively. n=2. (**E**) Protein levels of various proteins (input and after pulldown with p53 or EGFR) in HCT116 cells expressing WT and mutant p53 under untreated and DDX31 siRNA treated conditions. n=2. (**F**) Protein levels of SHP1 in cytosolic and nuclear fractions from HCT116 and H1299 cells expressing WT and mutant p53. n=2. (**G**) Protein levels of SHP1 pulled down with EGFR in cytosolic and nuclear fractions from HCT116 and H1299 cells expressing WT and mutant p53. n=2. Two-tailed unpaired Student's t-test was performed. (* $p<0.05$, ** $p<0.01$, *** $p<0.001$, **** $p<0.0001$). Source data are provided as a Source Data file.

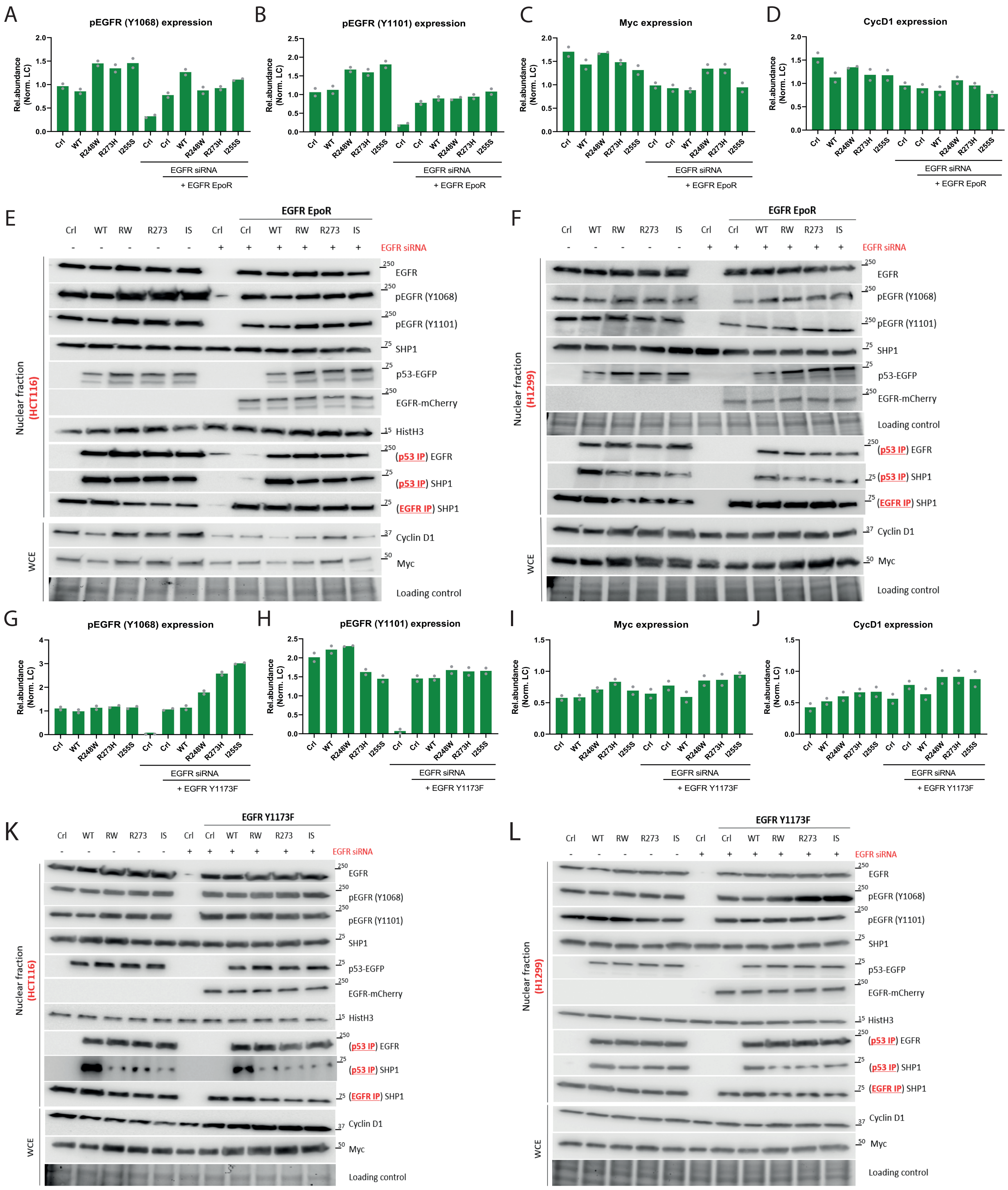
A
pEGFR (Y1068) expression
Rel.abundance (Norm. LC)
Crl
WT
R248W
R273H
I255S
EGFR siRNA
+ EGFR EpoR
B
pEGFR (Y1101) expression
C
Myc expression
D
CycD1 expression
E
EGFR EpoR
RW
R273
IS
EGFR siRNA
Nuclear fraction (HCT116)
EGFR
pEGFR (Y1068)
pEGFR (Y1101)
SHP1
p53-EGFP
EGFR-mCherry
HistH3
(p53 IP) EGFR
(p53 IP) SHP1
(EGFR IP) SHP1
WCE
Cyclin D1
Myc
Loading control
F
Nuclear fraction (H1299)
G
+ EGFR Y1173F
H
I
J
K
EGFR Y1173F
L

Supplementary Fig. 8: EGFR mutants validate DBD mutant effects on EGFR-SHP1 interaction

(**A-D**) Quantification of (A, B) pEGFR, (C) c-Myc and (D) Cyclin D1 protein levels in H1299 cells expressing DBD mutants. Cells were also treated with EGFR siRNA and expressed EGFR EpoR (high affinity for SHP1). n=2. (**E-F**) Protein levels of EGFR, pEGFR and SHP1 in input nuclear fractions and Cyclin D1 and c-Myc in whole-cell extract from HCT116 and H1299 cells expressing DBD mutants. Cells were also treated with EGFR siRNA and expressed EGFR EpoR (high affinity for SHP1). Protein levels of EGFR and SHP1 pulled down with p53 and/or EGFR. n=2. (**G-J**) Quantification of (G, H) pEGFR, (I) c-Myc and (J) Cyclin D1 protein levels in H1299 cells expressing DBD mutants. Cells were also treated with EGFR siRNA and expressed EGFR Y1173F (reduced affinity for SHP1). n=2. (**K-L**) Protein levels of EGFR, pEGFR and SHP1 in input nuclear fractions and Cyclin D1 and c-Myc in whole-cell extract from HCT116 and H1299 cells expressing DBD mutants. Cells were also treated with EGFR siRNA and expressed EGFR Y1173F (reduced affinity for SHP1). Protein levels of EGFR and SHP1 pulled down with p53 and/or EGFR. n=2. Source data are provided as a Source Data file.

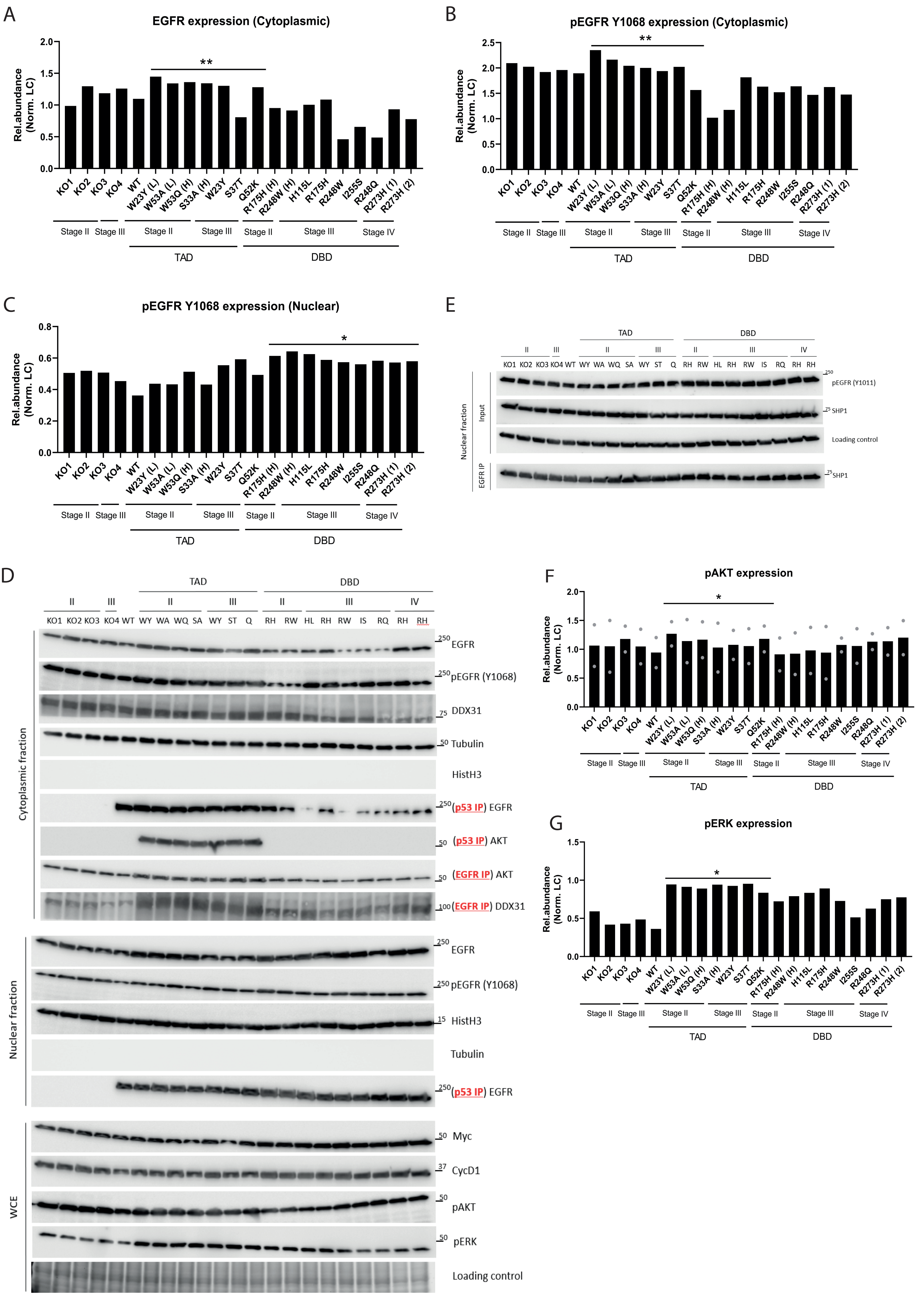
A
EGFR expression (Cytoplasmic)
Rel.abundance (Norm. LC)
B
pEGFR Y1068 expression (Cytoplasmic)
C
pEGFR Y1068 expression (Nuclear)
D
E
F
pAKT expression
G
pERK expression
KO1 KO2 KO3 KO4 WT W23Y (L) W53A (L) W53Q (H) S33A (H) W23Y S37T Q52K R175H (H) R248W (H) H115L R175H R248W I255S R248Q R273H (1) R273H (2)
Stage II Stage III Stage II Stage III Stage II Stage III Stage IV
TAD
DBD
Cytoplasmic fraction
Nuclear fraction
WCE
EGFR
pEGFR (Y1068)
DDX31
Tubulin
HistH3
(p53 IP) EGFR
(p53 IP) AKT
(EGFR IP) AKT
(EGFR IP) DDX31
Myc
CycD1
pAKT
pERK
Loading control
pEGFR (Y1011)
SHP1
Input
EGFR IP

Supplementary Fig. 9: Mechanistic validation of mutant p53 activation of EGFR signaling in patient tumors

(**A-C**) Quantification of (A) cytosolic EGFR and (B) cytoslic and (C) nuclear pEGFR (Y1068) protein levels in p53 null and TAD and DBD mutant patient colorectal tumors. (**D**) Protein levels of various factors in cytosolic and nuclear fractions from p53 null and TAD and DBD mutant patient colorectal tumors. HCT116 cells expressing WT p53 as a control. Protein levels of EGFR, AKT and DDX31 pulled down with p53 or EGFR. (**E**) Protein levels of pEGFR (Y1011) and SHP1 in nuclear fractions from p53 null and DBD mutant patient colorectal tumors. HCT116 cells expressing WT p53 as a control. Protein levels of SHP1 pulled down with EGFR. (**F-G**) Quantification of (F) pAKT and (G) pERK protein levels in p53 null and TAD and DBD mutant patient colorectal tumors. HCT116 cells expressing WT p53 as a control. All statistical tests performed on TAD (n=7 independent patients) versus DBD (n=9 independent patients). Two-tailed unpaired Student's t-test was performed. (* $p<0.05$, ** $p<0.01$, *** $p<0.001$, **** $p<0.0001$). Source data are provided as a Source Data file.

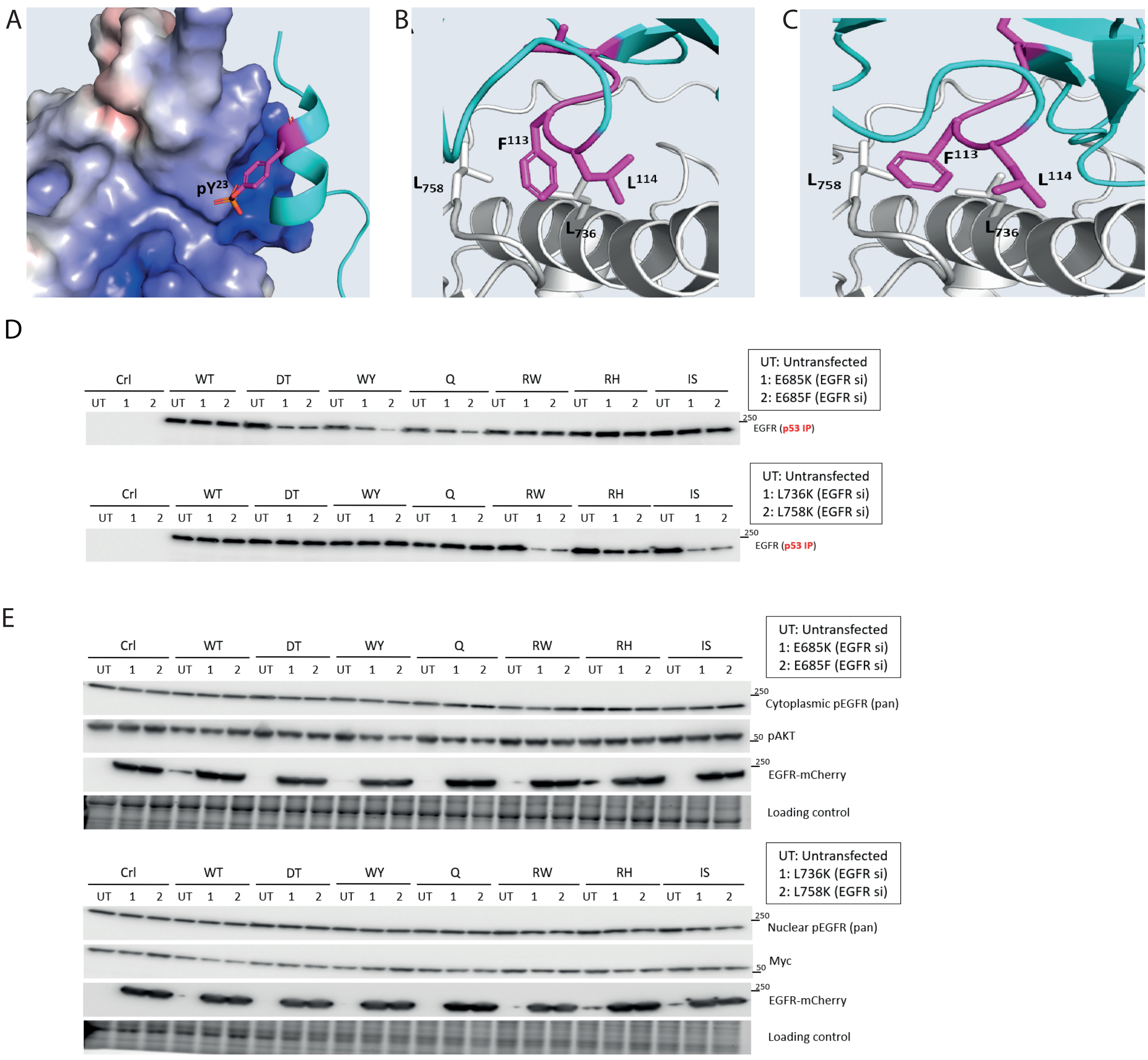

A
pY23
B
F113
L758
L114
L736
C
F113
L758
L114
L736
D
Crl WT DT WY Q RW RH IS
UT 1 2
UT: Untransfected
1: E685K (EGFR si)
2: E685F (EGFR si)
250
EGFR (p53 IP)
UT: Untransfected
1: L736K (EGFR si)
2: L758K (EGFR si)
250
EGFR (p53 IP)
E
UT: Untransfected
1: E685K (EGFR si)
2: E685F (EGFR si)
250
Cytoplasmic pEGFR (pan)
50
pAKT
250
EGFR-mCherry
Loading control
UT: Untransfected
1: L736K (EGFR si)
2: L758K (EGFR si)
250
Nuclear pEGFR (pan)
50
Myc
250
EGFR-mCherry
Loading control

Supplementary Fig. 10: Validation of structural EGFR mutants that disrupt interaction with mutant p53

(**A**) Structure of EGFR-p53TADW23Y complex. EGFR is shown with its surface colored according to the electrostatic potential (with red to blue color corresponding to -5kcal/mol to + 5kcal/mol) and the p53TAD1 peptide is shown as cartoon (Cyan). Residue pY23 (phosphorylated tyrosine 23) from TAD1 is shown as sticks. Phosphorylation status of W23Y mutant was predicted using the webserver NetPhos-3.1. (**B-C**) Structures of EGFR-p53DBDI255S and EGFR-p53DBDR248W complexes. EGFR is shown with its surface colored according to the electrostatic potential (with red to blue color corresponding to -5kcal/mol to + 5kcal/mol) and (B) p53DBD-I255S and (C) p53DBD-R248W shown as cartoon (Cyan) with the solvent exposed hydrophobic patch highlighted in magenta. Residues F113, L114 from p53 DBD and L736, L758 from EGFR shown as sticks. Disruption of hydrophobic interactions due to L736K and L758K mutations. (**D**) Protein levels of EGFR pulled down with p53 in HCT116 cells expressing WT and mutant p53. Cells were also treated with EGFR siRNA and expressed the EGFR mutants that disrupt TAD binding (E685K, E685F) and mutants that disrupt DBD binding (L736K, L758K). n=2. (**E**) Protein levels of pEGFR, pAKT and c-Myc in HCT116 cells expressing WT and mutant p53. Cells were also treated with EGFR siRNA and expressed the EGFR mutants that disrupt TAD binding (E685K, E685F) and mutants that disrupt DBD binding (L736K, L758K). n=2. Source data are provided as a Source Data file.

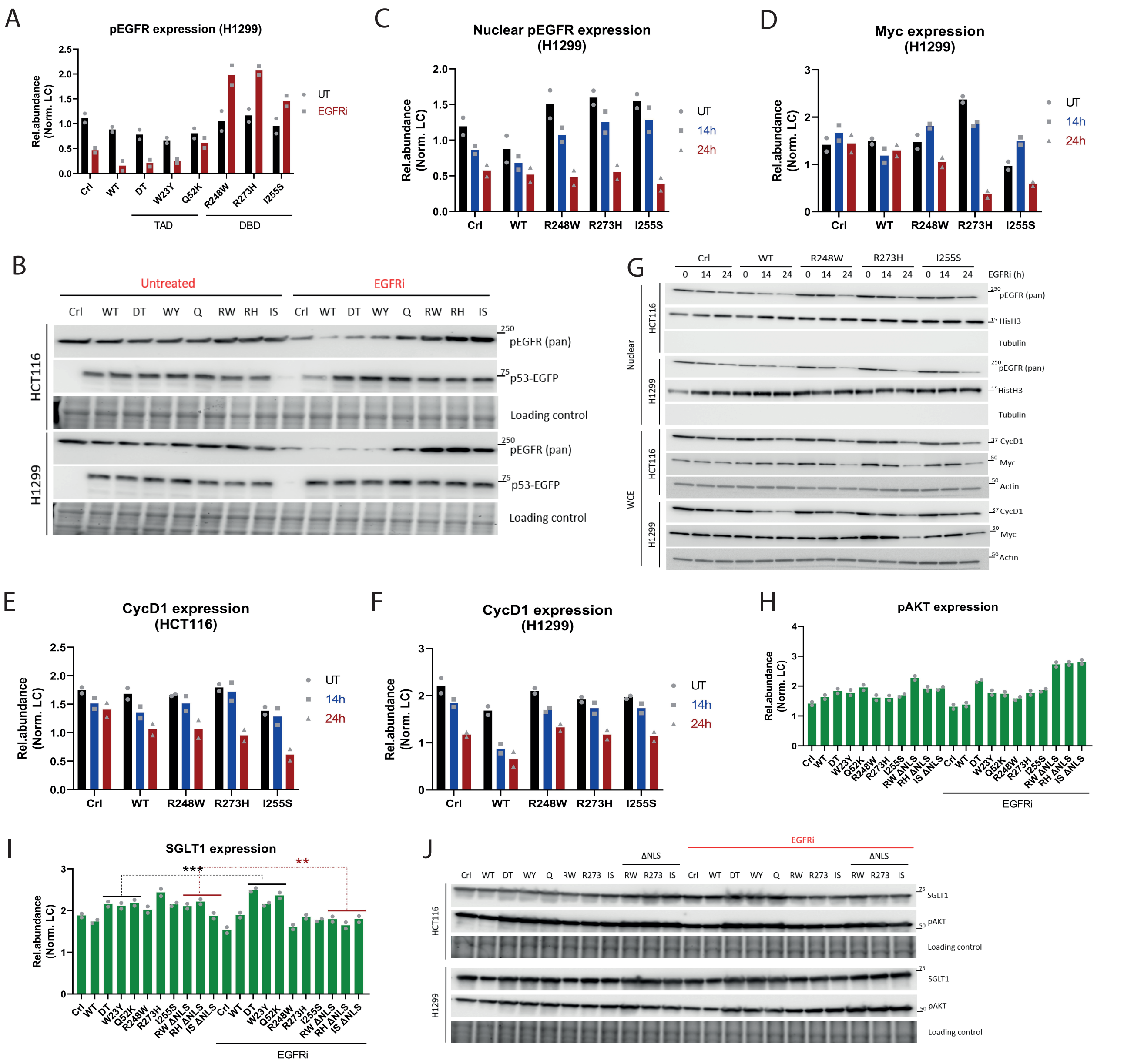

K

	Crl E685K	Crl E685F	WT E685K	WT E685F	DT E685K	DT E685F	WY E685K	WY E685F	Q E685K	Q E685F	RW E685K	RW E685F	RH E685K	RH E685F	IS E685K	IS E685F
IC50	1621	2077	1676	644.9	628.7	686.5	90.25	466.0	453.2	4809	47.55	8.324	9.381	24.07	31.32	5.117

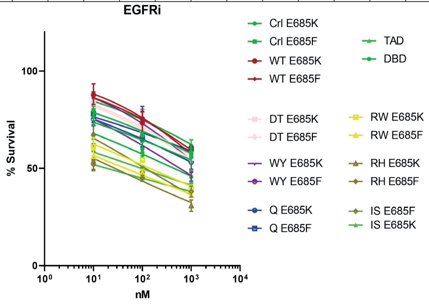

L

	Crl L736K	Crl L758K	WT L736K	WT L758K	DT L736K	DT L758K	WY L736K	WY L758K	Q L736K	Q L758K	RW L736K	RW L758K	RH L736K	RH L758K	IS L736K	IS L758K	TAD	DBD
IC50	4461	276.8	6911	39259	84.36	77.52	146.8	33.49	1486	171.5	5.520	8.708	2.524	0.9991	1.348	3.632	2364	84.52

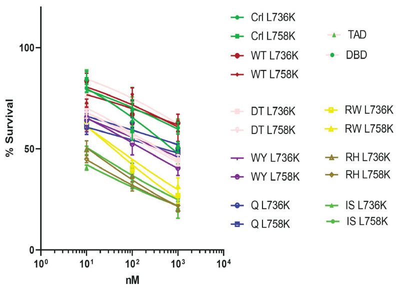

Supplementary Fig. 11: Mutant p53 confers resistance to EGFR inhibition via different mechanisms

(**A**) Quantification of pEGFR (pan) protein levels in H1299 cells expressing WT and mutant p53 treated with EGFR inhibitor. n=2. (**B**) Protein levels of pEGFR (pan) and p53-EGFP in HCT116 and H1299 cells expressing WT and mutant p53 treated with EGFR inhibitor. n=2. (**C-F**) Quantification of (C) nuclear pEGFR (pan), (D) whole-cell c-Myc in H1299 cells and (E,F) Cyclin D1 protein levels in HCT116 and H1299 cells expressing WT and mutant p53 treated with EGFR inhibitor. n=2. (**G**) Protein levels of nuclear pEGFR (pan), whole-cell c-Myc and Cyclin D1 in HCT116 and H1299 cells expressing WT and mutant p53 treated with EGFR inhibitor. n=2. (**H-I**) Quantification of (H) pAKT and (I) SGLT1 protein levels in H1299 cells expressing WT and mutant p53. Cells were also treated with EGFRi. Statistical tests performed on TAD (n=3 independent mutants) versus DBDΔNLS (n=3 independent mutants).n=2. (**J**) Protein levels of SGLT1 and pAKT in HCT116 and H1299 cells expressing WT and mutant p53. Cells were also treated with EGFRi. n=2. (**K-L**) Dose response curves of HCT116 cells expressing WT and mutant p53 treated with EGFR inhibitor. Cells were also treated with EGFR siRNA and expressed the EGFR mutants that disrupt (K) TAD binding (E685K, E685F) and mutants that disrupt (L) DBD binding (L736K, L758K). n=3 independent experiments. Two-tailed unpaired Student's t-test was performed. (* $p<0.05$, ** $p<0.01$, *** $p<0.001$, **** $p<0.0001$). Source data are provided as a Source Data file.

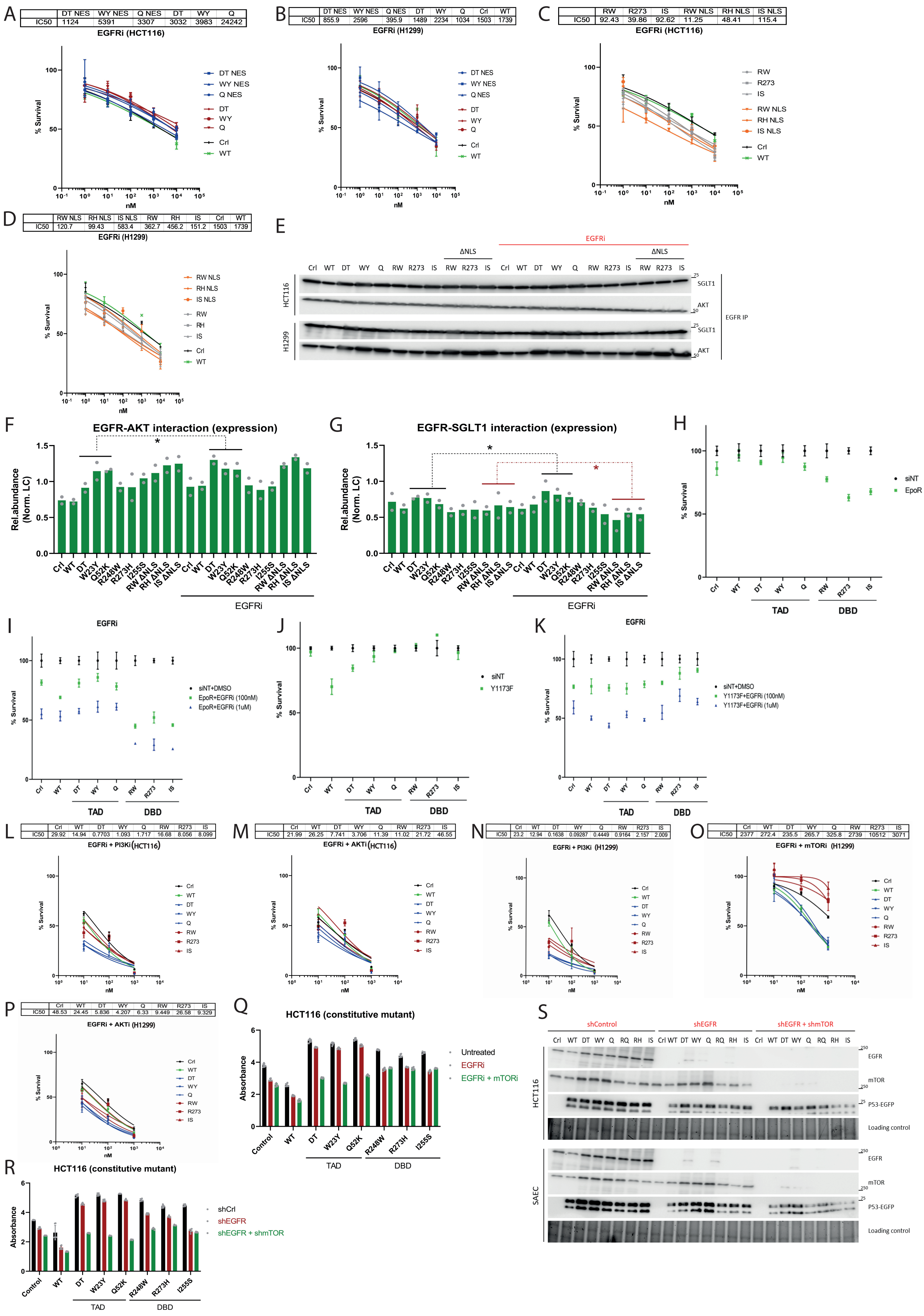
A
IC50 | DT NES 1124 | WY NES 5391 | Q NES 3307 | DT 3032 | WY 3983 | Q 24242
EGFRi (HCT116)
B
IC50 | DT NES 855.9 | WY NES 2596 | Q NES 395.9 | DT 1489 | WY 2234 | Q 1034 | Crl 1503 | WT 1739
EGFRi (H1299)
C
IC50 | RW 92.43 | R273 39.86 | IS 92.62 | RW NLS 11.25 | RH NLS 48.41 | IS NLS 115.4
EGFRi (HCT116)
D
IC50 | RW NLS 120.7 | RH NLS 99.43 | IS NLS 583.4 | RW 362.7 | RH 456.2 | IS 151.2 | Crl 1503 | WT 1739
EGFRi (H1299)
% Survival
nM
E
EGFRi
ΔNLS
HCT116
H1299
SGLT1
AKT
EGFR IP
F
EGFR-AKT interaction (expression)
Rel.abundance (Norm. LC)
G
EGFR-SGLT1 interaction (expression)
H
siNT
EpoR
TAD
DBD
I
EGFRi
siNT+DMSO
EpoR+EGFRi (100nM)
EpoR+EGFRi (1uM)
J
siNT
Y1173F
K
EGFRi
siNT+DMSO
Y1173F+EGFRi (100nM)
Y1173F+EGFRi (1uM)
L
IC50 | Crl 29.92 | WT 14.94 | DT 0.7703 | WY 1.093 | Q 1.717 | RW 16.68 | R273 8.056 | IS 8.099
EGFRi + PI3Ki (HCT116)
M
IC50 | Crl 21.99 | WT 26.25 | DT 7.741 | WY 3.706 | Q 11.39 | RW 11.02 | R273 21.72 | IS 46.55
EGFRi + AKTi (HCT116)
N
IC50 | Crl 23.2 | WT 12.94 | DT 0.1638 | WY 0.09287 | Q 0.4449 | RW 0.9164 | R273 2.157 | IS 2.009
EGFRi + PI3Ki (H1299)
O
IC50 | Crl 2377 | WT 272.4 | DT 235.5 | WY 265.7 | Q 325.8 | RW 2739 | R273 10512 | IS 3071
EGFRi + mTORi (H1299)
P
IC50 | Crl 48.53 | WT 24.45 | DT 5.836 | WY 4.207 | Q 6.33 | RW 9.449 | R273 26.58 | IS 9.329
EGFRi + AKTi (H1299)
Q
HCT116 (constitutive mutant)
Absorbance
Untreated
EGFRi
EGFRi + mTORi
R
HCT116 (constitutive mutant)
shCrl
shEGFR
shEGFR + shmTOR
S
shControl
shEGFR
shEGFR + shmTOR
HCT116
SAEC
EGFR
mTOR
P53-EGFP
Loading control

Supplementary Fig. 12: Mutant p53 confers resistance to EGFR inhibition via different mechanisms

(**A-D**) Dose response curves of (A, C) HCT116 and (B, D) H1299 cells expressing WT and mutant p53 treated with EGFR inhibitor. n=3 independent experiments. (**E**) Protein levels of SGLT1 and AKT in HCT116 and H1299 cells expressing WT and mutant p53 and treated with EGFRi. Protein levels of SGLT1 and AKT pulled down with EGFR. n=2. (**F-G**) Quantification of (F) AKT (after EGFR pulldown) and (G) SGLT1 (after EGFR pulldown) protein levels in HCT116 cells expressing WT and mutant p53 and treated with EGFRi. n=2. (**H-K**) Survival plots various cancer cell lines and HCT116 expressing WT and mutant p53 treated with EGFR inhibitor. Cells were treated with EGFR siRNA and expressed (H, I) EGFR EpoR (high affinity for SHP1) or (J, K) EGFR Y1173F (reduced affinity for SHP1). n=3 independent experiments. (**L-M**) Dose response curves of HCT116 cells expressing WT and mutant p53 treated with EGFR inhibitor in combination with (L) PI3Ki and (M) AKTi. n=3 independent experiments. (**N-P**) Dose response curves of H1299 cells expressing WT and mutant p53 treated with EGFR inhibitor in combination with (N) PI3Ki, (O) mTORi, (P) AKTi. n=3 independent experiments. (**Q-R**) Quantification of crystal violet intensity from colony formation assays using HCT116 cells constitutively expressing WT and mutant p53. Cells were treated with (Q) EGFRi or EGFRi+mTORi and (R) inducible expression of shControl or shEGFR and/or shmTOR. n=4 independent experiments. Error bars denote Mean ± SEM of technical replicates. $p<0.0001$ for DBD untreated vs DBD EGFRi/DBD shEGFR and TAD untreated vs TAD EGFRi+mTORi/shEGFR+shmTOR (n=3 mutants each). (**S**) Protein levels of EGFR, mTOR and p53-EGFP in HCT116 and SAECK cells constitutively expressing WT and mutant p53 and doxycycline-induced expression of shEGFR and shmTOR. Two-tailed unpaired Student's t-test was performed. (* $p<0.05$, ** $p<0.01$, *** $p<0.001$, **** $p<0.0001$). Source data are provided as a Source Data file.
