# Supplementary material for: Domain-specific p53 mutants activate EGFR by distinct mechanisms exposing tissue-independent therapeutic vulnerabilities
Source: Nat Commun. 2023 Mar 28;14:1726. doi: 10.1038/s41467-023-37223-3 (PMC10050071; doi:10.1038/s41467-023-37223-3)
Supplement: Supplementary file 2 — Description of Additional Supplementary Files [file 41467_2023_37223_MOESM2_ESM.pdf]

Description of Additional Supplementary Files

File Name: Supplementary Data 1
Description: Information on *TP53* mutational status and tumor Stage (including risk of metastatic or recurrent diseases for Stage II patients) of our patient cohort.
