# Supplementary material for: Domain-specific p53 mutants activate EGFR by distinct mechanisms exposing tissue-independent therapeutic vulnerabilities
Source: Nat Commun. 2023 Mar 28;14:1726. doi: 10.1038/s41467-023-37223-3 (PMC10050071; doi:10.1038/s41467-023-37223-3)
Supplement: Supplementary file 4 — Reporting Summary [file 41467_2023_37223_MOESM4_ESM.pdf]

nature portfolio

Corresponding author(s): Ashok R. Venkitaraman
Last updated by author(s): Mar 5, 2023

Reporting Summary

Nature Portfolio wishes to improve the reproducibility of the work that we publish. This form provides structure for consistency and transparency in reporting. For further information on Nature Portfolio policies, see our Editorial Policies and the Editorial Policy Checklist.

Statistics

For all statistical analyses, confirm that the following items are present in the figure legend, table legend, main text, or Methods section.

n/a	Confirmed	
☐	☒	The exact sample size (*n*) for each experimental group/condition, given as a discrete number and unit of measurement
☐	☒	A statement on whether measurements were taken from distinct samples or whether the same sample was measured repeatedly
☐	☒	The statistical test(s) used AND whether they are one- or two-sided<br>*Only common tests should be described solely by name; describe more complex techniques in the Methods section.*
☐	☒	A description of all covariates tested
☒	☐	A description of any assumptions or corrections, such as tests of normality and adjustment for multiple comparisons
☐	☒	A full description of the statistical parameters including central tendency (e.g. means) or other basic estimates (e.g. regression coefficient) AND variation (e.g. standard deviation) or associated estimates of uncertainty (e.g. confidence intervals)
☐	☒	For null hypothesis testing, the test statistic (e.g. *F*, *t*, *r*) with confidence intervals, effect sizes, degrees of freedom and *P* value noted<br>*Give P values as exact values whenever suitable.*
☒	☐	For Bayesian analysis, information on the choice of priors and Markov chain Monte Carlo settings
☒	☐	For hierarchical and complex designs, identification of the appropriate level for tests and full reporting of outcomes
☒	☐	Estimates of effect sizes (e.g. Cohen's *d*, Pearson's *r*), indicating how they were calculated

Our web collection on statistics for biologists contains articles on many of the points above.

Software and code

Policy information about availability of computer code

Data collection	Proteome Discoverer (v2.4), Mascot (v2.6.1), HADDOCK (v2.4)
Data analysis	Image J (v1.6), GraphPad Prism (v9), CFX Maestro Software (v2), R package gplots (v3), R LIMMA (v3), Reactome (v75)

For manuscripts utilizing custom algorithms or software that are central to the research but not yet described in published literature, software must be made available to editors and reviewers. We strongly encourage code deposition in a community repository (e.g. GitHub). See the Nature Portfolio guidelines for submitting code & software for further information.

Data

Policy information about availability of data

All manuscripts must include a data availability statement. This statement should provide the following information, where applicable:

- Accession codes, unique identifiers, or web links for publicly available datasets
- A description of any restrictions on data availability
- For clinical datasets or third party data, please ensure that the statement adheres to our policy

The raw mass spectra and search data have been uploaded to the jPOST repository with the following accession numbers: JPST001483 (jPOST) and PXD031725 (ProteomeXchange).
Public peptide databases such as SwePep (http://www.swepep.org/), Uniprot (https://www.uniprot.org/) and NeuroPep (http://isyslab.info/NeuroPep/) were used.

Human research participants

Policy information about studies involving human research participants and Sex and Gender in Research.

Reporting on sex and gender	NA
Population characteristics	NA
Recruitment	25 patients were consented
Ethics oversight	National University Hospital (NUH), Institutional Review Boards (IRBs) in Singapore

Note that full information on the approval of the study protocol must also be provided in the manuscript.

Field-specific reporting

Please select the one below that is the best fit for your research. If you are not sure, read the appropriate sections before making your selection.

☒ Life sciences ☐ Behavioural & social sciences ☐ Ecological, evolutionary & environmental sciences

For a reference copy of the document with all sections, see nature.com/documents/nr-reporting-summary-flat.pdf

Life sciences study design

All studies must disclose on these points even when the disclosure is negative.

Sample size	Patient sample size was based on obtaining consent for the approved period requested in ethics approval applications.<br>Sample size for various in vitro investigations (drug studies, western blot analyses) was based on having at least 3 TAD and DBD mutants.
Data exclusions	No data were excluded from the analysis.
Replication	Attempts at replication were successful (number of times stated in individual figure legends as n = x).
Randomization	Patients were grouped based on TP53 mutational status as determined by sequencing analyses.
Blinding	Blinding was not relevant as the TP53 mutational status of the patients and various cell lines needed to be known.

Reporting for specific materials, systems and methods

We require information from authors about some types of materials, experimental systems and methods used in many studies. Here, indicate whether each material, system or method listed is relevant to your study. If you are not sure if a list item applies to your research, read the appropriate section before selecting a response.

Materials & experimental systems

n/a	Involved in the study	
☐	☒	Antibodies
☐	☒	Eukaryotic cell lines
☒	☐	Palaeontology and archaeology
☐	☒	Animals and other organisms
☐	☒	Clinical data
☒	☐	Dual use research of concern

Methods

n/a	Involved in the study	
☒	☐	ChIP-seq
☒	☐	Flow cytometry
☒	☐	MRI-based neuroimaging

Antibodies

Antibodies used	anti-p53 (DO-1, Santa Cruz Biotechnology, SC-126), anti-EGFR (Cell Signaling, #4267), anti-pEGFR (Y1068) (Cell Signaling, #2234), anti-pEGFR (Y1101) (eBioscience, #EM1991), anti-AKT (Cell Signaling, #9272), anti-EGFP (Chromotek, #3H9-20), anti-mCherry (Invitrogen, #M11217), anti-pmTOR (Cell Signaling, #2971), anti-pAKT (Cell Signaling, #4060), anti-pS6K (Cell Signaling, #9204), anti-pERK (Cell Signaling, #4370), anti-CycD1 (Abcam, ab226977), anti-cMyc (Cell Signaling, #5605), anti-E2F1 (Cell Signaling, #3742), anti-SGLT1 (Santa Cruz Biotechnology, SC-20582), anti-SHP1 (Cell Signaling, #3759), anti-DDX31 (Cell Signaling, #8761), anti-actin (Abcam, ab8227), anti-tubulin (Abcam, ab7291) and anti-histone H3 (Santa Cruz Biotechnology, SC-517576), anti-γH2AX (Millipore, #05-636), anti-Ki67 (Invitrogen, #MAS-14520)

Validation	Validation as noted on manufacturer's webpage and spec sheets and data presented in manuscript.

Eukaryotic cell lines

Policy information about cell lines and Sex and Gender in Research

Cell line source(s)	Cell lines were purchased from ATCC.
Authentication	Cell lines were either purchased from ATCC (authenticated) or authenticated by STR profiling.
Mycoplasma contamination	All cell lines used tested negative for mycoplasma.
Commonly misidentified lines (See ICLAC register)	NA

Animals and other research organisms

Policy information about studies involving animals; ARRIVE guidelines recommended for reporting animal research, and Sex and Gender in Research

Laboratory animals	4 month old male and female NOD-scid IL2Rgammanull (NSG) mice
Wild animals	This study did not involve wild animals.
Reporting on sex	Such information was not collected as not relevant.
Field-collected samples	This study did not involve samples collected from the field.
Ethics oversight	A*STAR Biological Resource Center

Note that full information on the approval of the study protocol must also be provided in the manuscript.

Clinical data

Policy information about clinical studies

All manuscripts should comply with the ICMJE guidelines for publication of clinical research and a completed CONSORT checklist must be included with all submissions.

Clinical trial registration	Institutional Review Boards (IRBs) in Singapore (DSRB Ref: 2018/01168)
Study protocol	NA as patient material was collected as a one-off.
Data collection	Patients were consented within a 6 month period as approved by ethics board.
Outcomes	NA
